# Characterization of a Novel Mannose-Binding Lectin with Antiviral Activities from Red Alga, *Grateloupia chiangii*

**DOI:** 10.3390/biom10020333

**Published:** 2020-02-19

**Authors:** Hyun-Ju Hwang, Jin-Wook Han, Hancheol Jeon, Kichul Cho, Ju-hee Kim, Dae-Sung Lee, Jong Won Han

**Affiliations:** 1 Department of Applied Bioresource Science, National Marine Biodiversity Institute of Korea, Seocheon 33662, Korea; hjhwang@mabik.re.kr (H.-J.H.); hiclow@mabik.re.kr (J.-W.H.); hjeon@mabik.re.kr (H.J.); win0830ner@naver.com (K.C.); 2 Department of Ecology and Conservation, National Marine Biodiversity Institute of Korea, Seocheon 33662, Korea; algae2030@mabik.re.kr; 3 Department of Genetic Resources Research, National Marine Biodiversity Institute of Korea, Seocheon 33662, Korea; daesung@mabik.re.kr

**Keywords:** lectin, *Grateloupia*, algae, hemagglutination, antiviral

## Abstract

Lectins have the ability to bind specific carbohydrates and they have potential applications as medical and pharmacological agents. The unique structure and usefulness of red algal lectin have been reported, but these lectins are limited to a few marine algal groups. In this study, a novel mannose-binding lectin from *Grateloupia chiangii* (*G. chiangii* lectin, GCL) was purified using antiviral screens and affinity chromatography. We characterized the molecular weight, agglutination activity, hemagglutination activity, and heat stability of GCL. To determine the carbohydrate specificity, a glycan microarray was performed. GCL showed strong binding affinity for Maltohexaose-β-Sp1 and Maltoheptaose-β-Sp1 with weak affinity for other monosaccharides and preferred binding to high-mannan structures. The N-terminal sequence and peptide sequence of GCL were determined using an Edman degradation method and LC-MS/MS, and the cDNA and peptide sequences were deduced. GCL was shown to consist of 231 amino acids (24.9 kDa) and the N-terminus methionine was eliminated after translation. GCL possessed a tandem repeat structure of six domains, similar to the other red algal lectins. The mannose binding properties and tandem repeat structure of GCL may confer it the potential to act as an antiviral agent for protection against viral infection.

## 1. Introduction

Lectin, discovered more than 100 years ago, is defined as a carbohydrate binding protein [1]. Lectin has the ability to agglutinate erythrocytes by recognizing specific carbohydrate chains on the surface of the cells, also referred to as agglutinins or hemagglutinins [2]. Lectins are widely distributed throughout bacteria, plants, and animals. They act as defensive molecules in plants by recognizing pathogenic organisms [3]. It has also been suggested that lectin is a defensive substance innate to the immune system of vertebrates and invertebrates [4]. Furthermore, lectin is related to a variety of other biological processes such as cell development, cell–cell interactions, and signaling pathways [5].

Because of the carbohydrate binding properties of lectins and their involvement in the defensive system and immune response of organisms, lectin has seen diverse applications in biological research and pharmacology [6,7,8]. For instance, the fluorescent lectins, combined with several other techniques, have been widely used to analyze the surface carbohydrates of cells or organelles [9]. Also, lectin immobilized matrices for glycoprotein isolation have become a common tool in glycobiology [10] and lectin micro-arrays have become popular tools for cell carbohydrate profiling [11]. Within the last decade, pharmacological applications of lectins have been reported [12], including those with anti-cancer (or anti-tumor) and anti-viral effects [13,14]. Because of lectins’ ability to specifically recognize cell surface carbohydrates, it has potential to grant specificity to drug delivery systems or to aid in diagnoses [15].

Algal lectins exhibit unique carbohydrate specificity and physico-chemical characteristics when compared to other plant lectins [16]. Lectins, including algal lectin, are able to bind and recognize a wide range of pathogens, including fungi, bacteria, viruses, and parasites, leading to agglutination and neutralization of these microorganisms [17]. For this reason, algal lectins have long been believed to have extraordinary potential for medical applications such as anti-tumor, anti-viral, and anti-microbial effects [16,17].

Algal lectins have been found in the green, brown, and red algal groups. Approximately 60% of algal lectins have come from Rhodophyta (red algae) [16] and the biomedical potential of lectins from these algae has been reported [17]. To date, about 500 lectins from red algae have been screened, but less than 40 lectins have been purified and sequenced [18]. Among the purified red algal lectins, few have had their pharmacological applications discussed or confirmed (i.e., Griffithsin) [19,20]. Although the role of lectins in red algae remains unclear, several roles have been suggested, including an involvement in spermatia and tricogyne mutual recognition in sexual reproduction [21,22].

Several red algal lectins possess a unique repeated primary structure [18] and the contribution of this tandem repeat structure to lectin activity was elucidated by recombinant technology [18,23]. These studies revealed that the primary structure of red algal lectin contributes to the production of lectin by recombinant proteins. Therefore, more information on red algal lectin is needed for deeper study. Among the diverse array of lectins, mannose-binding lectin (belonging to the collectin, or C-type pattern recognition lectins) is a popular choice for studies that focus on antivirals or viral infection pathways. The relevance of mannose receptors in viral infections has been reported [13,24], and implies the importance of mannose-binding lectins in the development of antiviral agents. Mannose-binding lectin was able to interrupt the self-assembly of viruses during viral replication [25], and has risen as a potential candidate for anti-viral agents in the form of high-dose mannose-binding lectin therapy against Ebola [26].

Griffithsin from *Griffithsia* sp. is a good example of a red algal lectin with therapeutic potential. Since the discovery of Griffithsin by Watson and Waaland [27], this protein has been widely studied with thousands of articles being published on it [28], putting red algal lectin in the spotlight. Griffithsin has specificity for mannose and possesses antiviral activity against HIV-1 [19,28] and Hepatitis C viral infections [29]. Although there are many reports that suggest the therapeutic potential of algal lectin, few lectins have had their biomedical properties and biological functions elucidated because of limited quantities or information. Thus, the accumulation of biological information for a variety of lectins is necessary.

In this study, a novel red algal lectin from *Grateloupia chianggi* was purified and partially characterized. Additionally, preliminary studies on the antiviral activity of *G. chianggi* lectin (GCL) were performed, leading to the discussion of potential applications for *G. chianggi* lectin in biochemical and medical research.

## 2. Materials and Methods

### 2.1. Algal Sources

Red alga *G. chianggi* was collected from the southern coast of Korea. Collected samples were cleaned twice with autoclaved sea-water and moisture was removed by a paper towel. The cleaned samples were stored at −80 °C until use.

### 2.2. Purification of GCL

The crude extract was prepared according to previous methods [30]. An algal sample (30 g) was immersed in liquid nitrogen and ground to a fine powder with a mortar and pestle. Five volumes of extraction buffer (Tris-buffered saline (TBS): 20 mM Tris-Cl, 150 mM NaCl, pH 7.5) were added to the sample to prepare the crude extract. The sample was incubated for 2 h at 4 °C, centrifuged at 20,000× *g* for 20 min at 4 °C and then the supernatant was collected as the crude extract. Then, D-mannose (Man) chromatography was immediately performed on the crude extract using a Bio-rad fast protein liquid chromatography system (Bio-rad, Berkeley, CA, USA). The column was washed with 10 volumes of TBS. Mannose-binding proteins were eluted with 0.5 M D-mannose with an extraction buffer by monitoring the absorbance at 280 nm. The fractions showing single bands following sodium dodecyl sulfate–polyacrylamide gel electrophoresis (SDS-PAGE) were fooled. The purified protein was dialyzed in TBS buffer overnight with buffer changes every 4 h. The total protein and purified protein concentrations were measured by a Bradford micro-assay [31] using an enzyme-linked immunosorbent assay (ELISA) reader (Epoch microplate spectrophotometer, BioTek, Winooski, VT, USA).

### 2.3. Partial Characterization of Lectin

The presence of inter- and intra-molecular disulfide bonds was determined by SDS-PAGE with the absence or presence of reductant DTT (1,4-dithiothreitol) in sample buffer. Protein stability at various temperatures was measured following previous methods [30]. The purified lectin was divided into 500 µL aliquots in microtubes. The water bath for testing was set to seven different temperatures, 30 °C, 40 °C, 50 °C, 60 °C, 70 °C, 80 °C, and 90 °C. Samples stored at room temperature were used as control. Samples were incubated at the designated temperature for 30 min, then removed and cooled to room temperature, followed by centrifugation at 12,000 *g* for 10 min to remove the insoluble materials produced during incubation. The supernatant was collected and used immediately in hemagglutination assays. The effect of divalent metal ions was determined by adding 5 mM MgCl_2_ and CaCl_2_, or the absence of divalent metal ions in the protein solution.

### 2.4. Hemagglutination Assay and Carbohydrate Specificity

Horse and sheep blood for the hemagglutination assay were purchased from Hanil Comed (Sungnam, Gyeonggi-do, Korea). Blood was washed with phosphate buffered saline (PBS, pH 7.3) until the red color of the supernatant disappeared. Erythrocytes were prepared to a 4% suspension in PBS. The lectin samples were serially diluted in a 96-well U bottom plate and then the 4% erythrocyte suspension was added to each well. After incubation at room temperature for 30 min, hemagglutination activity was judged.

Carbohydrate specificity was measured by a hemagglutination inhibition test. The following carbohydrates and glycoproteins were used for the inhibition test; D-glucose (Glc), D-mannose, D-galactose (Gal), *N*-acetyl-D-glucosamine (GlcNAc), *N*-acetyl-D-galactosamine, L-fucose, fructose, lactose, and bovine fetuin. Samples (25 µL) that have four hemagglutination activities, were mixed with each carbohydrate (25 µL) and 25 µL of mixture was removed from the well. An equal volume of 4% horse erythrocyte suspension was added to the sample and mixed. The plates were incubated at room temperature for 30 min and inhibition was measured.

### 2.5. Determination of N-Terminal Amino Acid Sequences

N-terminal amino acid sequences were determined by the Korea Basic Science Institute (KBSI, Seoul, Korea). Protein bands were transferred to polyvinylidene difluoride membranes using a Western blot kit (Bio-rad, USA). Membranes were stained with ponsiue S staining solution. Single bands on the membrane were excised using a knife and sent to KBSI. N-terminal sequencing was performed using a Procise 491 HT protein sequencer (Applied Biosystems).

### 2.6. Peptide Mapping Using Mass Spectrometry

Peptide mapping was performed according to previous methods [23]. Protein bands obtained from SDS-PAGE were excised, in-gel digested with trypsin, and cleaned with Zip-Tip (Millipore, Billerica, MA, USA). Mass spectrometry analyses were performed using Capillary LC-Nano ESI-MS with a 6545 Q-TOF LC/MS (Agilent Technologies, Santa Clara, CA, USA). Samples were applied to a ZORBAX 300SB-C8 column (1 × 50 mm, 3.5 μm; Agilent) equilibrated with 0.1% (*v*/*v*) formic acid in mass grade water and eluted by a gradient between water and 100% acetonitrile at a flow rate of 10 µL/min. The tuning parameters used for mass analyses were as follows: capillary temperature 300 °C, source voltage 1.9 kV, skimmer voltage 45 V, and fragmentor voltage 175 V.

### 2.7. Cloning of GCL and Determination of cDNA Sequences

Based on the N-terminal sequencing and peptide mapping results, the cDNA sequence was screened with transcriptome data (generated by Hi-seq 3000). The full cDNA sequence was confirmed by PCR. Total RNA was obtained using a Qiagen Plant total RNA isolation kit following the manufacture’s protocol. The quality of total RNA was determined using a spectrophotometer and formaldehyde agarose gel electrophoresis. First strand cDNA was synthesized with oligo-dT primers using a cDNA synthesis kit from Promega (Madison, WI, USA). cDNA was purified using an Intron PCR purification kit and immediately used for PCR. PCR primers were designed based on transcriptome data and N-terminal sequencing results (Table 1). The PCR reaction was performed as follows: 95 °C 2 min for pre-denaturation, 35 cycles of 95 °C for 20 s, 60 °C for 30 s, 72 °C for 1 min, and then the final reaction was performed at 72 °C for 10 min. The PCR product was loaded onto agarose gel for electrophoresis and the target band was excised with a sharp knife. The PCR product was purified using a gel elution kit following the manufacture’s protocol. The isolated PCR product was cloned into a T-easy cloning vector and transformed into host DH5α. The transformant was plated on a Luria-Bertani agar plate containing 100 µg/mL of ampicillin and incubated at 37 °C overnight. After incubation, positive colonies were collected and cultured in Luria-Bertani broth at 37 °C overnight.

Plasmids were isolated using a plasmid isolation kit (Qiagen, Hilden, Germany). The DNA was sequenced by the Sanger based method (Macrogen, Seoul, Korea). 

### 2.8. Glycan Microarray

A glycan microarray analysis was performed by Ebiogen (Seoul, Korea) according to previous methods [23]. The Glycan Array kit was purchased from RayBioTech (Norcross, GA, USA). An array containing 300 synthetic glycans printed in quadruplicate on a glass slide was used. Label-based detection was performed according to the manufacturer’s protocols and previous methods [23]. Biotinylated recombinant lectins and native lectins at 50 μg/mL were added to the array wells and incubated for >3 h with gentle rocking. The glass slide was washed with 1× wash buffer I and II, provided in the kit. Glycan-lectin binding was detected by incubation with Cy3 equivalent dye-conjugated streptavidin for 1 h at room temperature. For cyanine-3 detection, the signals were visualized using a microarray laser scanner (GenePix 4100A; Molecular Devices, Sunnyvale, CA, USA) with excitation at 554 nm and emission at 568 nm. Data extraction was performed using the microarray analysis software, GenePix Pro (ver. 7.2, Sunnyvale, CA, USA). Glycan array data were normalized and analyzed using RayBio Analysis software (RayBioTech, GA-Glycan-300-SW, Norcross, GA, USA).

### 2.9. Antiviral Activity Test

Antiviral assays were performed at the Center for Convergent Research of Emerging Virus Infection at the Korea Research Institute of Chemical Technology (Daejeon, Korea) following previous protocols [32].

Madin-Darby canine kidney (MDCK) cells from the American Type Culture Collection (ATCC, Manassas, VA, USA), African green monkey kidney Vero cells (ATCC), and HTLV-1-infected human T lymphocyte MT-4 cells were used for antiviral assays against the influenza virus, herpes simplex virus (HSV), and human immunodeficiency virus (HIV), respectively. MDCK cells were grown in minimum essential medium (Gibco/Invitrogen, Carlsbad, CA, USA) supplemented with 10% fetal bovine serum (Invitrogen) at 37 °C. Vero cells were grown at 37 °C in Dulbecco’s modified Eagle’s medium (Gibco/Invitrogen) supplemented with 10% fetal bovine serum (Invitrogen).

Influenza virus strains, A/Puerto Rico/8/34 (H1N1) (PR8), A/Hong Kong/8/68 (H3N2) (HK), and B/Lee/1940 (B) were obtained from ATCC and propagated in 10-day-old chicken embryos at 37 °C (PR8 and HK) or MDCK cells at 35 °C (B) for 3 days. For the anti-HSV assay, the HSV1 strain F and HSV strain MS were used. HIV1 strain IIb and HIV2 strain ROD was used for the anti-HIV assay. Antiviral assays were performed based on the virus-induced cytopathic effect (CPE reduction assay).

In the anti-influenza viral assay, MDCK cells were seeded onto 96-well plates and either mock-infected or infected with influenza virus at a multiplicity of infection of 0.001 (50 plaque-forming units of influenza virus per well). After incubation for 1 h at 35 °C (PR8, BB, and HK), the medium was removed, and GCL was added and subsequently serially diluted in minimum essential medium containing TPCK (L-1-Tosylamide-2-phenylethyl chloromethyl ketone)-trypsin (Sigma). On day 2 or 3 post-infection, cell viability was analyzed with an MTT [3-(4,5-dimethylthiazol-2-yl)-2,5-diphenyltetrazolium bromide] assay. In the anti-HSV assay, HSV infected or non-infected Vero cells were incubated for 3 days and then cell viability was measured by an MTT assay. In the anti-HIV assay, MT-4 cells were centrifuged and the supernatant was removed, and the harvested cells were infected by HIV. Mock-infected cells were added with RPMI 1640/10% in place of HIV as a control. Cells were diluted to 1 x 10^5^ cells/mL. The cells were incubated at 37 °C in a CO_2_ incubator for 5 days. Cell viability was measured by an MTT assay.

The 50% cytotoxic concentration (CC_50_) and the 50% effective concentration (EC_50_) values were calculated using SoftMax Pro Software (Pro 7, Molecular Devices, Sunnyvale, CA, USA).

## 3. Results

### 3.1. Purification of Mannose-Binding Lectin

**Figure 1:**
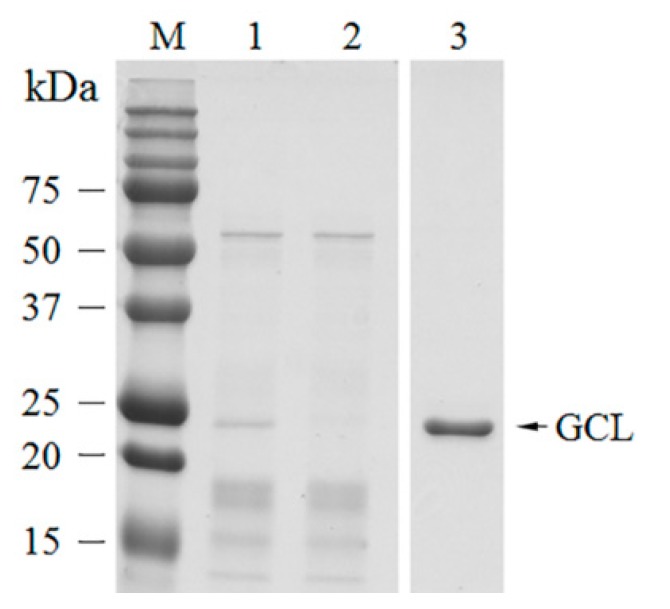
Purification of *Grateloupia chianggi* lectin (GCL) using D-mannose affinity chromatography. M = molecular weight marker; 1 = crude extract lane; 2 = flow-through fraction lane; 3 = purified GCL lane.

GCL was successfully purified using mannose affinity chromatography. Single protein bands were observed from isolated fractions without any impurities (Figure 1). The molecular weight of GCL was ~25 kDa as determined by SDS-PAGE (Figure 1). Approximately 13.8 mg of protein were extracted from 30 g of *G. chiangii* (wet weight). A total of 0.65 mg of GCL was obtained from 30 g of *G. chiangii,* accounting for 4.7% of all proteins and the specific agglutination activity was 51,200 (titer/mg). Purification increased 14.72-fold (Table 2).

### 3.2. Molecular Properties and Cloning of GCL

The sequence of the N-terminal amino acids of GCL was determined to be Val-Val-Ser-Asn-Arg-Lue-Val-Ser-Gly-Glu-X-Leu-His-Arg from Edman degradation analysis. The full-length cDNA sequence was determined by PCR combined with transcriptome data generated by a Hi-seq 2000 sequencer. The cDNA consisted of 900 bp including 696 bp of open reading frame. The calculated molecular weight of the protein was 24.9 kDa and the theoretical isoelectric point was p*I* 6.97, which corresponded to SDS-PAGE data. In total, mass data from nine peptides were obtained from LC-MS/MS data that covered about 70% of the protein sequence. The peptide mapping data and N-terminal sequencing result were consistent with the predicted peptide mass. The N-terminal methionine was removed by post transcriptional modification according to comparisons between data from the intact N-terminal sequence (Figure S1).

Interestingly, GCL possessed a tandem repeat structure that contained six repeat domains of ~30-mer (Figure S2). GCL was determined to be a member of the B class lectins and possessed similarity to bacterial mannose-binding lectins. The mannose binding sites and dimerization interface were well conserved (Figure S3). However, similar proteins from plants or algae could not be identified using a GenBank database.

### 3.3. Hemagglutination Activity and Carbohydrate Specificity

**Figure 2:**
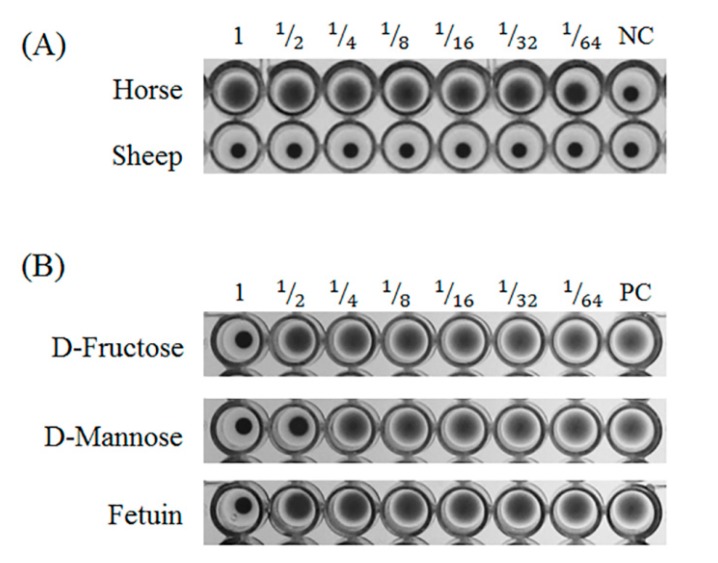
Hemagglutination assay and carbohydrate specificity of *Grateloupia chianggi* lectin (GCL). (**A**) Hemagglutination activity of GCL (25 μg/mL) in horse and sheep blood. (**B**) Carbohydrate specificity of GCL (four hemagglutination activity units of GCL were used for the inhibition test). A serial two-fold dilution was obtained (left to right); NC, negative control; PC, positive control.

GCL agglutinated horse erythrocytes but not sheep erythrocytes. The minimum concentration of GCL for agglutination of horse erythrocytes was 0.8 µg/mL (Figure 2A).

Inhibition experiments were performed with ten different carbohydrates to determine specificity. The hemagglutination activity of GCL was inhibited by the monosaccharides, D-mannose and fructose at 125 mM and 250 mM, respectively. The hemagglutination activity was also inhibited by the glycoprotein fetuin at 195 μg/mL. The other carbohydrates tested were unable to inhibit activity (Figure 2B, Table 3).

### 3.4. Effect of Temperature and Divalent Ions on GCL

**Figure 3:**
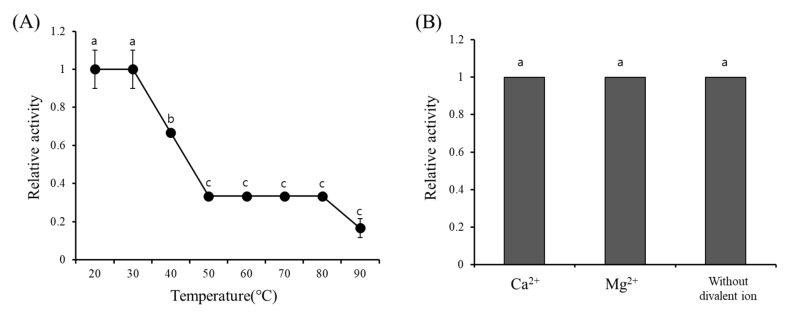
Activity of *Grateloupia chianggi* lectin (GCL) at various temperatures and in the presence of divalent ions. (**A**) Effect of temperatures on GCL activity. (**B**) Effect of the presence or absence of divalent ions, n = 3. Error bars represent mean ± standard deviation of triplicate experiments and different letters indicate significant difference (*p* < 0.05).

Heat stability of GCL was measured at various temperatures (20–90 °C). GCL activity was not affected until 30 °C. Half of activity was lost at 40 °C. Interestingly, about 15% of the original activity was maintained even after being subjected to 90 °C heat for 30 min (Figure 3A). The activity of GCL was not significantly affected by the presence of divalent cations as the addition of the divalent cations, Mg^2+^ or Ca^2+^, did not increase the GCL activity (Figure 3B).

### 3.5. Glycan Micro-Array

**Figure 4:**
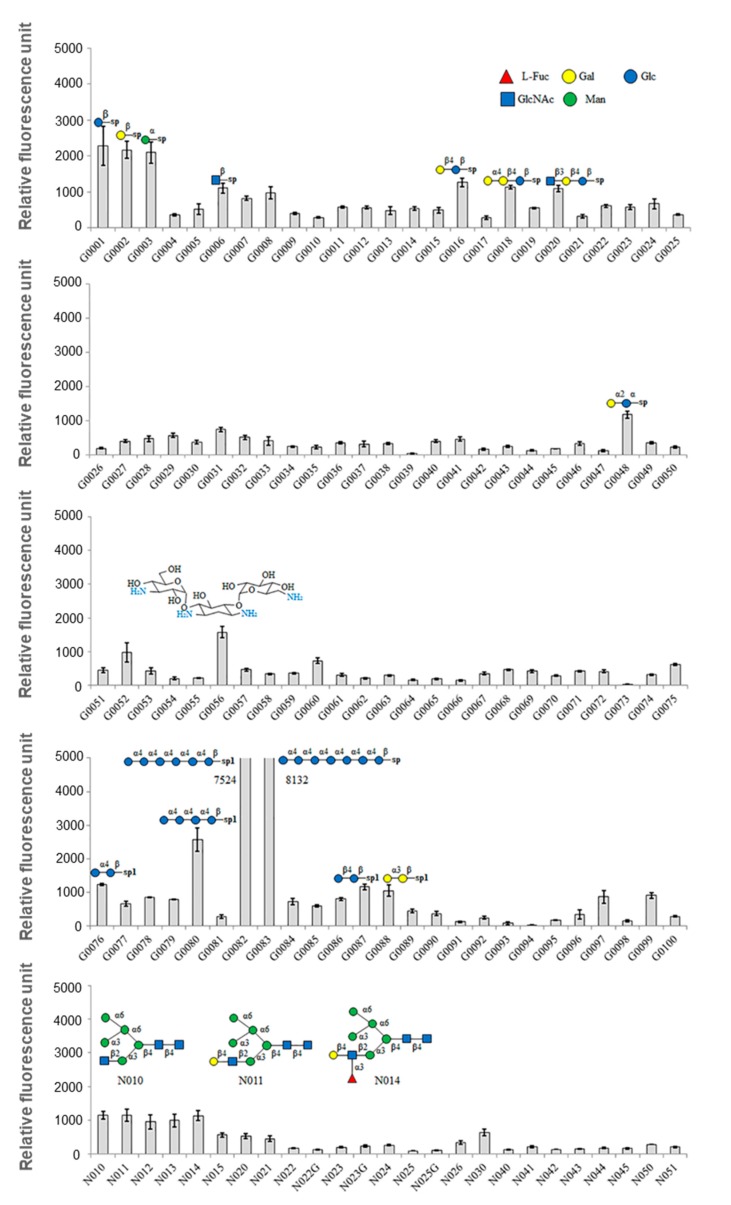
Glycan array of *Grateloupia chianggi* lectin. Relative fluorescence units were calculated using an array analysis program (RayBioTech). The glycan structure is given for signals that exceeded 1000 relative fluorescence units. Error bars represent mean ± standard deviation (SD) of triplicate experiments.

In order to determine the carbohydrate specificity, a glycan micro-array was performed which immobilized 300 different carbohydrates (Table S1). Positive signals over 1000 were observed in 18 of the 300 tested carbohydrates (Figure 4, Table 4). GCL demonstrated weak binding affinity for the monosaccharides, β-Glc-sp, β-Gal-sp, and α-Man-sp with normalized signal intensities of 2579, 2269, and 2194, respectively. GLC also exhibited binding affinity for Maltotetraose-β-Sp1 with a signal intensity of 2705. GCL possessed a strong binding affinity for Maltohexaose-β-Sp1 and Maltoheptaose-β-Sp1. The protein also exhibited weak binding affinity for N-glycan (signal intensity of 1000). The high-mannan structures, i.e., Man-α-1,6-(Man-α-1,3-)Man-α-1,6-(GlcNAc-β-1,2-Man-α-1,3-)Man-β-1,4-GlcNAc-β-1,4-GlcNAc-Sp5 were also shown to interact with GCL (Figure 4).

### 3.6. Antiviral Activity of GCL

The antiviral activity of GCL was screened against the influenza virus (PR8, HK, and Lee type), HIV type 1, and herpes (HSV1 and HSV2). GCL was effective against the influenza virus and HSV (Table 5, Table 6) but was not effective against HIV. Influenza infection was successfully inhibited by GCL treatment at 1.37 μM (H1N1), 0.95 μM (H3N2), and 1.05 μM (Flu B) (Table 5). The antiviral effect was more pronounced against HSV, with an EC_50_ of 0.0001–0.0152 μM of GCL (Table 6). However, GCL did not inhibit HIV infection at any concentration tested. GCL showed no toxicity to cell lines at the tested concentrations. 

## 4. Discussion

A novel D-mannose-binding lectin (GCL) from the red alga, *G. chiangii*, was successfully purified and molecularly characterized. In the current literature, many red algal lectins have been screened by hemagglutination tests but few have been purified and molecularly characterized. In the present study, mannose affinity chromatography was a powerful tool for the purification of GCL from whole cell extracts. Approximately 5 mg of protein were obtained from 10 g of plants. The yield of lectin from *G. chiangii* was moderate compared to other algal lectins, such as that from *Aglaothamnion callophyllidicola* [21].

GCL was identified as a mannose-binding lectin, with a molecular weight of ~25 kDa by SDS-PAGE. The estimated molecular weight corresponded to molecular weights calculated from the deduced amino acid sequences. The N-terminal amino acid sequence determined by Edman degradation exactly matched the deduced amino acid sequence. The N-terminal methionine of GCL was eliminated during post-modification of the protein. N-terminal methionine excision is a conserved pathway in all compartments where protein synthesis occurs [33]. About 70% of peptide fragments that were discovered by MS/MS spectrometry were covered by the protein sequence.

Interestingly, GCL showed an absence of cysteine residues in its amino acid sequence, suggesting that GCL is a monomeric protein rather than a multimeric protein. GCL possessed a tandem repeat structure which consisted of six domains. Generally, the monomeric form of red algal lectin structures is distinguishable from land plants or animals, which are multimeric and are connected by disulfide bonds or other chemical interactions [21]. Similar to other red algal lectins that have short peptide (~30-mer) tandem repeat structures [18], GCL possessed a repeat structure in its primary structure. This may contribute to the stability of lectin’s tertiary structure and may be a substitute for the advantages conferred through multimeric structures [18,34]. The tandem repeat structure of GCL was considered to be preferable for our purposes, as this is shared with other red algal lectins and may contribute to an increase in hemagglutination activity [18,23,34]. Furthermore, the isolated GCL amino acid sequence interestingly represented high similarity with bacterial lectins as shown in Supplementary Figure S4. We considered these results are derived from the lack of sequence information concerning red algal lectins in the NCBI database. Also, whereas prokaryotic lectins usually not contain poly-A sequences, the eukaryotic lectins represent poly-A amino acid sequences as shown in Figure S1, and thus we considered GCL is an algal lectin.

The hemagglutination activity of GCL did not require divalent ions. It was also shown that GCL possessed no heat tolerance, which may be caused by a lack of cysteine residues, even though 20% of activity remained after exposure to 90 °C for 30 min. The temperature range for heat stability of red algal lectins is wide, varying from 30 to 100 °C [17], with most falling between 30 and 50 °C [17]. In the case of *Gracilaria ornata* lectin, activity was not lost until 50 °C [35]. *G. ornata* lectin is made up of at least 7.79% cysteine residues while GCL possesses no cysteine. Lack of cysteine residues results in hydrogen or ionic interactions in protein structures being easily broken, but intra-molecular disulfide bonds may retain structural stability through covalent bonds in *G. ornata* lectin [35].

The isolated GCL specifically agglutinated horse blood cells, but not sheep blood cells. Isolated GCL was inhibited by D-mannose and fructose as well as the glycoprotein, fetuin. However, as hemagglutination inhibition tests do not reflect diverse glycans, a glycan array was used to determine the glycan recognition properties of GCL. Among 300 glycans, 18 glycans demonstrated a positive signal over 1000 RFU. Similar to the inhibition test results, GCL showed specificity toward α-Man. In contrast to the inhibition test, binding properties differed for the alpha (α-) and beta (β-) conformations of sugars, i.e., glucose and galactose. Only α-conformation monosaccharides were used in the inhibition test, and as such, it can be assumed that GCL demonstrates stereoisomerism with regard to the sugar-lectin binding. With the exception of a few red algal lectins such as the lectins from *Hypnea cervicornis* [36], *Hypnea musciformis* [37] and *Vidalia obtusiloba* [38], most lectins are specific for glycoproteins such as mucin and fetuin. The glycan composition and validated proteoforms of bovine fetuin is previously well-described by comparing with human fetuin and recombinant human fetuin [39].

Glycoprotein specific lectins are more complicated to use in research or medical applications than those that are specific to monosaccharides because of the difficulty in predicting their reactivity. GCL was able to bind not only to monosaccharides but also to glycoproteins. The previous review has reported that a lot of algal lectins, especially from red algae, commonly have distinct characteristics compared to those of terrestrial plants such as low molecular weight, monomeric form, high thermostability, an affinity for glycoproteins but not for monosaccharides, and no divalent cations requirements [40]. However, interestingly, GCL represented affinities for specific monosaccharide including D-mannose and fructose (Table 3). Similar to GCL, monosaccharide-binding lectins are also reported from several algae such as *Enteromorpha* sp. [41] and *Ulva* sp. [42]. Also, the previous report showed that HIV inhibiting algal lectins usually exhibit high mannose-binding property. However, no HIV-inhibiting activity was observed from purified GCL. Based on the results, we concluded the GCL is novel algal lectins showing distinct nature than the other reported algal lectins.

GCL possessed the highest specificity for Maltotetraose-β, Maltohexaose-β, and Maltoheptaose-β. As the length of the sugar chain increased, the signal likewise increased. The reason behind this difference in signal intensity is not straightforward, but the assumption is that a greater number of GCLs were bound to these longer chains rather than there being a difference in the binding strength between lectin and sugars as the length of the sugar chain increases.

Additionally, GCL could bind to high-mannan N-glycans that are related to antiviral abilities. With this sugar specificity, GCL’s antiviral activity could be assumed because the antiviral activities of other mannose-binding lectins have already been reported [43]. In addition to these antiviral properties, anti-cancer properties of mannose-binding lectins have also been reported in marine algae [16,17].

The antiviral effect have been reported by lectins derived from diverse organisms including prokaryotic organisms (*Microcystis viridis* lectin, cyanovirin-N, actinohivin, scytovirin, and etc.), plants (Jacalin, BanLec, Griffithsin, *Scilla campanulata* lectin, *Narcissus pseudonarcissus* lectin, *Galanthus nivalis* agglutinin, and etc.), and marine eukaryotic organisms (*Chaetopterus variopedatus* lectin, *Serpula vermicularis* lectin, *Crenomytilus grayanus* lectin, and etc.) [13]. The mechanism of antiviral activity of lectins has been well-described by previous review [13]. According to the report, a lot of lectins derived from bacteria, plants, and marine algae effectively suppress the viral replication by reacting with envelope glycoproteins [13]. A common viral recognition and entry regulated by a specific protein called “glycosylated envelope proteins (GEP).” A GEP usually has affinity for cell-surface proteins of host cells, and existing antiviral lectins react with high-mannose glycan structures, and thus trigger post-translational modifications of viral GEP. For instance, a GEP complex of HIV (Env complex) is composed of the transmembrane trimer of gp31 and the extracellular trimer of gp120 containing N-linked oligosaccharide attachment sites. These structures assist viral evasion of the host immune system and entry into the host cells mediated by recognition of CD4^+^ triggering a cascade of conformational rearrangement of Env complex. The antiviral lectins inhibit conformational reorganization of the Env complex, and thus lead to suppressed viral fusion into host cells [13].

Our preliminary tests demonstrated an antiviral activity of GCL against the influenza virus and HSV but not against HIV. GCL was more effective against HSV than the influenza virus, requiring only 1 to 20 nM to inhibit HSV infection. The antiviral activities of 12 different lectins were tested and the ESA-2 lectin had an EC_50_ of 12.4 nM [44,45]. ESA-2 specifically recognizes high mannose N-glycans, similarly to GCL. Inhibition of viral infection by ESA-2 is intermediated by blocking viral binding to a critical portion of the target cell, because certain high mannose glycan(s) are present on the region of the virus surface involved in receptor binding [40,41]. GCL had antiviral activities 100 times (1.37 μM) lower than ESA-2 and AAL [44,45]. Although GCL was less effective against influenza than ESA-2, GCL represented comparable values to the positive controls, RBV and AMT. GCL was also able to bind to high mannose N-glycans, thus we hypothesize that it may inhibit viral infections by similar mechanisms of ESA-2. However, the elucidation of these mechanisms will require comprehensive studies on GCL and viral interactions.

In this study, a novel mannose-binding lectin from the red alga, *G. chiangii,* was obtained and termed GCL. The antiviral activity of this lectin was tested and was determined to be a prospective antiviral agent.

## 5. Conclusions

Mannose-binding lectin from the red alga, *G. chiangii* was purified and termed GCL. GCL specifically bound the monosaccharides, β-Glc-sp, β-Gal-sp, and α-Man-sp, and possessed a strong affinity for Maltohexaose-β-Sp1 and Maltoheptaose-β-Sp1. High-mannan structures such as Man-α-1,6-(Man-α-1,3-)Man-α-1,6-(GlcNAc-β-1,2-Man-α-1,3-)Man-β-1,4-GlcNAc-β-1,4-GlcNAc-Sp5 also interact with GCL. Based on this carbohydrate specificity, an antiviral property of GCL was suggested, and indeed, purified lectin had an antiviral activity against the influenza virus and HSV.

## Figures and Tables

**Table 1:** Primers for amplification of *Grateloupia chianggi* lectin (GCL).

Name	Sequence (5’-3’)	Purpose
GCL-1	GGNGARTGYYTNCAYCG	
GCL-2	GGNGARTGYYTNCAYAG	
GCL-3	GTNTCNGGNGARTGYCT	
GCL-4	GTNAGYGGNGARTGYCT	
GCL-5	GTNGTNTCNAAYCGNCT	
GCL-6	GTNGTNTCNAAYCGNTT	
GCL-7	GTNGTNTCNAAYAGTCT	
GCL-8	GTNGTNTCNAAYAGTRTT	
DPP	GGTGAATGCTGCGACTACGATCCCCCTTTTTTTTTTTTTTTTTT	
GCL-F	ATGGTTGTCTCCAACAGAC	For the full length GCL gene
GCL-R	CGTATTGGTAGCCCAG	For the full length GCL gene

**Table 2:** Purification of *Grateloupia chianggi* lectin (GCL) from 30 g of *G. chiangii.* Hemagglutination activity was determined using horse erythrocytes.

Condition	Total Protein (mg)	Concentration (mg/mL)	Total Activity (Titer)	Specific Activity (Titer/mg)	Percentage of Recovery	Purification Fold
Crude Extract	13.8	0.092	48,000	3478	100	1.00
Affinity Chromatography	0.65	0.025	33,280	51,200	69.3	14.72

**Table 3:** Minimum inhibitory concentration of lectin by treatment of various substances.

Substance	Concentration (mM)
D-Glucose	NI *
D-Galactose	NI
D-Mannose	125
N-Acetyl-D-Glucosamine	NI
N-Acetyl-D-Galactosamine	NI
L-Fucose	NI
Maltose	NI
Lactose	NI
Fructose	250
Fetuin	195 **

* NI, absence of inhibition at 250 mM. ** 195 µg/mL (due to unidentified molecular weight).

**Table 4:** Overview of carbohydrate structures recognized by *Grateloupia chianggi* lectin (GCL).

No.	Glycan Structure	RFU (Normalized)
		GCL
**Monosaccharides**		
G0001	β-Glc-Sp	2273 ± 544
G0002	β-Gal-Sp	2165 ± 237
G0003	α-Man-Sp	2093 ± 294
G0006	β-GlcNAc-Sp	1104 ± 133
Disaccharides		
G0016	Gal-β-1,4-Glc-β-Sp	1260 ± 126
G0048	Glc-α-1,2-Gal-α-Sp	1173 ± 110
G0076	Glc-α-1,4-Glc-β-Sp1	1238 ± 28
G0087	D-cellulose-β-Sp1	1165 ± 82
Globo series, Milk Oligosaccharides, and GAGs		
G0018	Gal-α-1,4-Gal-β-1,4-Glc-β-Sp	1124 ± 47
G0020	GalNAc-β-1,3-Gal-β-1,4-Glc-β-Sp	1092 ± 92
Amino Glycoside		
G0056	Sisomicin sulfate	1583 ± 177
Natural Oligosaccharides		
G0080	Maltotetraose-β-Sp1	2577 ± 351
G0082	Maltohexaose-β-Sp1	7524 ± 1976
G0083	Maltoheptaose-β-Sp1	8132 ± 1810
N-glycans		
N-010	Man-α-1,6-(Man-α-1,3-)Man-α-1,6-(GlcNAc-β-1,2-Man-α-1,3-)Man-β-1,4-GlcNAc-β-1,4-GlcNAc-Sp5	1142 ± 116
N-011	Man-α-1,6-(Man-α-1,3-)Man-α-1,6-(Gal-β-1,4-GlcNAc-β-1,2-Man-α-1,3-)Man-β-1,4-GlcNAc-β-1,4-GlcNAc-Sp5	1148 ± 173
N-014	Man-α-1,6-(Man-α-1,3-)Man-α-1,6-[Gal-β-1,4-(Fuc-α-1,3-)GlcNAc-β-1,2-Man-α-1,3-]Man-β-1,4-GlcNAc-β-1,4-GlcNAc-Sp5	1129 ± 151
Human Milk Oligosaccharides		
H0400	Gal-β-1,4-Glc-Sp	1044 ± 110

Glc = glucose; Gal = galactose; Man = mannose; GlcNAc = N-acetyl-D-glucosamine; GalNAc = N-acetyl-D-galactosamine; Fuc = Fucose.

**Table 5:** Antiviral activity of *Grateloupia chianggi* lectin (GCL) against influenza virus strains.

Compounds	CC50 (μM) in MDCK Cells	EC_50_ (μM) Against Influenza Viruses (SI)		
		Flu A		Flu B
		Puerto Rico/8/34 (H1N1) (PR8)	Hong Kong/8/68 (H3N2) (HK)	Lee/1940 (B)
GCL	>1.6 ^£^	1.37 ± 0.31 (ND)	0.95 ± 0.09 (ND)	1.05 ± 0.2 (ND)
OSV-C	>100.0	0.071 ± 0.01 (>1409.7)	<0.004 ± 0.0004 (>26,657.4)	1.83 ± 0.18 (>54.5)
AMT	>100.0	>100.0 (ND)	13.2 ± 3.5 (>7.5)	>100.0 (ND)
RBV	>100.0	18.88 ± 2.17 (>5.3)	19.22 ± 3.89 (>4.6)	18.14 ± 0.71 (>138.1)

^£^ Maximum concentration of GCL that could be prepared. SI = selectivity index [ratio of CC50 (50% cytotoxic concentration) to EC_50_ (50% effective concentration)]; ND = not determined; AMT = amantadine hydrochloride; RBV = ribavirin; OSV-C, = oseltamivir carboxylate.

**Table 6:** Antiviral activity of *Grateloupia chianggi* lectin (GCL) against herpes simplex virus (HSV).

	Toxicity CC_50_	Antiviral Activity (EC_50_), μM		Selective Index	
		HSV1 (F)	HSV2 (MS)	HSV1 (F)	HSV2 (MS)
GCL	>1.6	0.0152	0.00144	>1.12	>5.6
Acyclovir	>100	0.52 ^£^	2.87 ^£^	>192	>35
Penosan polysulfate	>100	4.77	2.10	21	>48

^£^ Maximum concentration of GCL that could be prepared.

## Supplementary Materials

The supplementary materials are available online at https://www.mdpi.com/2218-273X/10/2/333/s1.

## Abbreviations

GCL	G. chianggi lectin
TBS	Tris-buffered saline
Man	D-mannose
DTT	1,4-Dithiothreitol
PBS	phosphate buffered saline
Glc	D-glucose
Gal	D-galactose
GlcNAc	*N*-acetyl-D-glucosamine
MDCK	Madin-Darby canine kidney
ATCC	American Type Culture Collection
ATCC	African green monkey kidney Vero cells
HSV	herpes simplex virus
HIV	human immunodeficiency virus
PR8	A/Puerto Rico/8/34
HK	A/Hong Kong/8/68
TPCK	L-1-Tosylamide-2-phenylethyl chloromethyl ketone-trypsin
MTT	3-(4,5-dimethylthiazol-2-yl)-2,5-diphenyltetrazolium bromide
CC_50_	The 50% cytotoxic concentration
EC_50_	the 50% effective concentration
GEP	glycosylated envelope proteins
