# Supplementary material for: Characterization of a Novel Mannose-Binding Lectin with Antiviral Activities from Red Alga, Grateloupia chiangii
Source: Biomolecules. 2020 Feb 19;10(2):333. doi: 10.3390/biom10020333 (PMC7072537; doi:10.3390/biom10020333)
Supplement: Supplementary file 1 [file biomolecules-10-00333-s001.pdf]

(A)

```
CTCTATATAACAAGCGCCAGTAATCCGGTTGGGATCACAGCCCAGTCATCTCAGACATCAACCACACTAGTTTTCCATTG 80

                                                N-terminal sequencing
AAAATTGAAATCTCTCTAACTTGTGGTGTTAACGCAAAGAAACATGGTTGTCTCCAACAGACTTGTCAGCGGTGAAAGTT 160
                                             M  V  V  S  N  R  L  V  S  G  E  S
                                                                           Pep-1
TGCATCGTGGACAACATATAATCTCGTCTGATCAGCATTTTATGTTGGTGCATCAATATGATGGGAATGTGGTCCTTTAC 240
L  H  R  G  Q  H  I  I  S  S  D  Q  H  F  M  L  V  H  Q  Y  D  G  N  V  V  L  Y

GATGTTGCCGGGAGTGGCTCAGGGAAACCTTTATGGAGCACGAACACATATGGTTCTCACACCACTGTCTTTAAAATGCA 320
 D  V  A  G  S  G  S  G  K  P  L  W  S  T  N  T  Y  G  S  H  T  T  V  F  K  M  Q

AGAGGATTCAAATCTCGTGCTTTATGCCACTGGTGGGAAACCGATATGGGCATCTGACACCCACGGTTCCGGTGGCAGGT 400
 E  D  S  N  L  V  L  Y  A  T  G  G  K  P  I  W  A  S  D  T  H  G  S  G  G  R
             Pep-2                                        Pep-3
TCCTGGAGATGCAAAATGATGGAAATTTGGTTGTGTACACCGACGTAGGTAAACCGAAATGGGCCTCGGACACCCATCAA 480
F  L  E  M  Q  N  D  G  N  L  V  V  Y  T  D  V  G  K  P  K  W  A  S  D  T  H  Q
            Pep-4                                                 Pep-5
GGGATTGCACCTCGCAGTGAAATCTCGGTGAATCAAGTCCTTCGCATACGCGAAGGGCTGCATTCTGCGAATAAGAGGAA 560
 G  I  A  P  R  S  E  I  S  V  N  Q  V  L  R  I  R  E  G  L  H  S  A  N  K  R  N
                     Pep-6
CGAGTTGATCTTGCAGAATGACGGAAATTTGGTTCTTTCTAAGAATGGAAGCACAGCCTGGAGTTCTGGAACTGCTGGTA 640
 E  L  I  L  Q  N  D  G  N  L  V  L  S  K  N  G  S  T  A  W  S  S  G  T  A  G
            Pep-7
CGGGGGCTAATGTTCTGGTCCTACAGATTGATGGCAACTTGGTGCTTTACAACGATACCAAGAGCGTCTGGGCTTCTGGT 720
T  G  A  N  V  L  V  L  Q  I  D  G  N  L  V  L  Y  N  D  T  K  S  V  W  A  S  G
                                                                       Pep-8
TCGATGAATAAGGGCGGTCGCACCGCGGTGATGCAGGACGACGGCAACTTTGTGCTTTACGCGGAGGGAGGAAAACCCGT 800
 S  M  N  K  G  G  R  T  A  V  M  Q  D  D  G  N  F  V  L  Y  A  E  G  G  K  P  V

CTGGGCTACCAATACGTAATACCGCTTCGATGCCGTTCCAGATATGTTCATTCACTAATGAAAGTGATGGGATAATCGAT 880
 W  A  T  N  T  *

TTACCACGCATATGTAGAAGTGCTTAAAAGAGCTTTCCCTAGAGTTGCAACTTGGCGTATGTGAGGATGCACGTACAGCA 960

TACCATAATATATGGTATACGTGCATCCTCACGGCAATACTACAGTATTGCTGTGTATGCGTAAAGCTTCCAGTAAAAAA 1040

ATCATTGAATTAGCAAAAAAAAAAAAAAAAAAA                                                1073
```

(B)

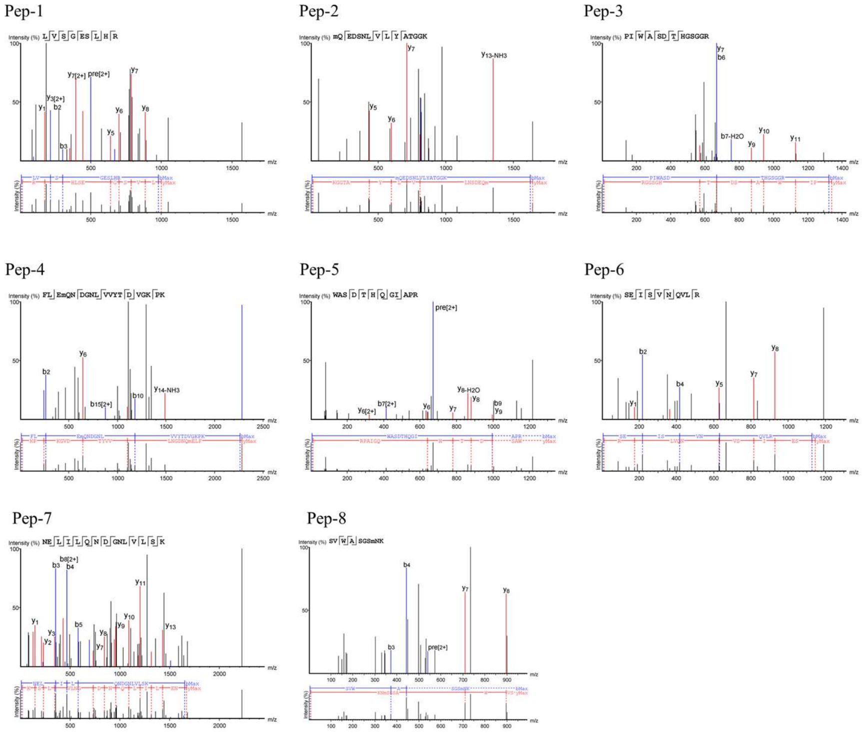

Figure 1. cDNA and amino acid sequence of *Glateloupia chianggi* lectin (GCL). (A) Deduced GCL cDNA and amino acid sequences. N-terminal sequence information is boxed. The peptide sequences from LC-MS/MS are underlined. (B) Peptide sequences (Pep-1-8).

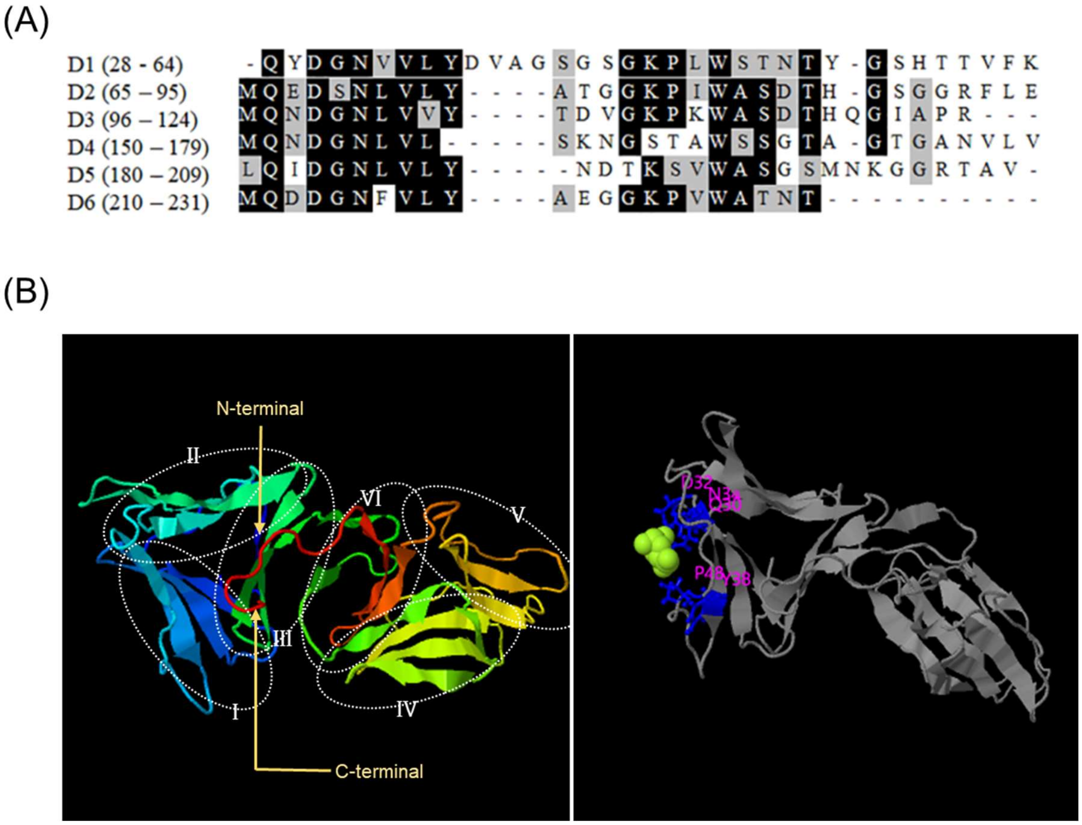

Figure 2. Tandem repeat structure of *Grateloupia chianggi* lectin. (A) Multiple alignment of tandem repeats. Black boxes indicate identical amino acids. (B) Schematic structural diagram of repeat domains (left side, I ~ IV), and ligand binding site (right side).

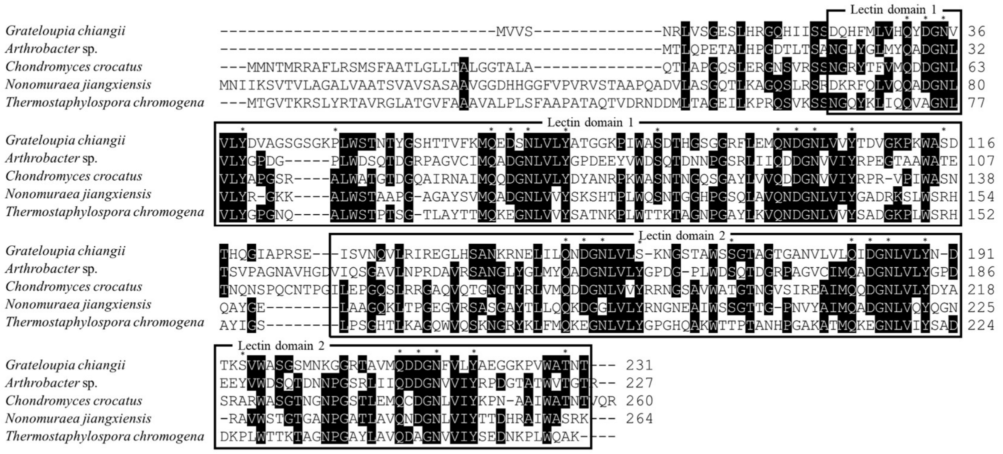

Figure 3. Multiple sequence alignment of GCL with related proteins from bacteria. The sequence alignment was generated by ClustalW alignment tool. Identical amino acids of the sequences are shaded in black. Predicted lectin domains are shown with boxes. The sequences were obtained from NCBI databank. *Grateloupia changii* (this study), *Arthrobacter* sp. (WP024366858), *Chondromyces crocatus* (AKT42434), *Nonomuraea jiangxiensis* (SDL12571), *Thermostaphylospora chromogena* (SDQ57287). Asterisks indicate ligand binding sites.

Table 1. Glycan microarray of native *Grateloupia chianggi* lectin (GCL), with binding signals normalized using a program provided by RayBioTech.

Substrate	Glycan structure	Normalized RFU	
		Mean	SD
**POS1**	–	5,687	0
**NEG**	-	599	174
**G0001**	β-Glc-Sp	2,273	545
**G0002**	β-Gal-Sp	2,165	237
**G0003**	α-Man-Sp	2,093	295
**G0004**	α-Fuc-Sp	353	32
**G0005**	α-Rha-Sp	516	157
**G0006**	β-GlcNAc-Sp	1,104	134
**G0007**	β-GalNAc-Sp	820	60
**G0008**	Tobramycin	972	160
**G0009**	Gal-β-1,3-GlcNAc-β-Sp	397	37
**G0010**	Gal-α-1,3-Gal-β-1,3-GlcNAc-β-Sp	287	16
**G0011**	Neu5Ac-α-2,3-Gal-β-1,3-GlcNAc-β-Sp	571	34
**G0012**	Neu5Ac-α-2,6-Gal-β-1,3-GlcNAc-β-Sp	562	39
**G0013**	Neu5Gc-α-2,3-Gal-β-1,3-GlcNAc-β-Sp	478	108
**G0014**	Neu5Gc-α-2,6-Gal-β-1,3-GlcNAc-β-Sp	533	55
**G0015**	Gal-β-1,3-(Fuc-α-1,4)-GlcNAc-β-[Lewis A]-Sp	492	81
**G0016**	Gal-β-1,4-Glc-β-Sp	1,260	127
**G0017**	Gal-α-1,3-Gal-β-1,4-Glc-β-Sp	276	52
**G0018**	Gal-α-1,4-Gal-β-1,4-Glc-β-Sp	1,124	47
**G0019**	GlcNAc-β-1,3-Gal-β-1,4-Glc-β-Sp	546	27
**G0020**	GalNAc-β-1,3-Gal-β-1,4-Glc-β-Sp	1,092	92
**G0021**	Neu5Ac-α-2,3-Gal-β-1,4-Glc-β-Sp	315	45
**G0022**	Neu5Ac-α-2,6-Gal-β-1,4-Glc-β-Sp	606	48
**G0023**	Neu5Gc-α-2,3-Gal-β-1,4-Glc-β-Sp	579	74
**G0024**	Neu5Ac-α-2,6-Gal-β-1,4-Glc-β-Sp	665	139
**G0025**	Gal-β-1,4-(Fuc-α-1,3)-Glc-β-Sp	365	15
**G0026**	GalNAc-β-1,3-Gal-α-1,4-Gal-β-1,4-Glc-β-Sp	186	22
**G0027**	GlcNAc-β-1,6-GlcNAc-β-Sp	396	41
**G0028**	4-P-GlcNAc-b-1,4-Man-b-Sp	464	77
**G0029**	Glc-α-1,2-Gal-α-1,3-Glc-α-Sp	563	63
**G0030**	Gal-β-1,3-GalNAc-α-Sp	370	52
**G0031**	Gal-β-1,4-GlcNAc-β-Sp	735	59
**G0032**	Gal-β-1,4-(Fuc-α-1,3)-GlcNAc-β-[Lewis X]–Sp	509	64
**G0033**	Neu5Ac-α-2,3-Gal-β-1,4-(Fuc-α-1,3)-GlcNAc-β-[sialyl Lewis X]-Sp	400	123
**G0034**	Neu5Ac-α-2,3-Gal-β-1,3-(Fuc-α-1,4)-GlcNAc-β-[sialyl Lewis A]-Sp	234	21
**G0035**	Neu5Gc-α-2,3-Gal-β-1,3-(Fuc-α-1,4)-GlcNAc-β-[sialyl Lewis A]-Sp	216	52
**G0036**	Gal-α-1,4-Gal-β-1,3-GlcNAc-β-Sp	355	32
**G0037**	Gal-β-1,4-GlcNAc-β-1,3-Gal-β-1,4-Glc-β-[LNnT]-Sp	311	86

**G0038**	GlcA-β-1,4-GlcNAc-α-1,4-GlcA-β-Sp	321	37
**G0039**	GlcNAc-β-1,6-(Gal-β-1,3)-GalNAc-α-O-Ser-Sp4	39	13
**G0040**	Neu5Ac-α-2,3Gal-β-1,4-(6S)GlcNAc-β-Sp	397	32
**G0041**	GalNAc-β-1,4-GlcNAc-β-Sp2	458	55
**G0042**	Neu5Ac-α-2,8-Neu5Ac-α-2,3-Gal β-1,4-Glc-β-Sp	161	34
**G0043**	Neu5Gc-α-2,8-Neu5Ac-α-2,3-Gal-β-1,4-Glc-β-Sp	234	28
**G0044**	GalNAc-α-1,3-(Fuc-α-1,2)-Gal-β-1,4-Glc-β-[blood A antigen tetrose]-Sp1	123	19
**G0045**	GlcNAc-β-1,2-Man-α-Sp	171	6
**G0046**	Neu5Ac-α-2,3-Gal-β-Sp1	320	53
**G0047**	Gal-β-1,3-GalNAc-β-1,3-Gal-β-Sp1	119	28
**G0048**	Glc-α-1,2-Gal-α-Sp	1,173	111
**G0049**	Gal-β-1,4-(Fuc-α-1,3)-GlcNAc-β-1,3-Gal-β-Sp1	342	36
**G0050**	Neu5Ac-α-2,3-Gal-β-1,4-(Fuc-α-1,3)-Glc-β-[3-sialyl-3-fucosyllactose/F-SL]-Sp1	223	31
**G0051**	GlcNAc-β-1,4-GlcNAc-β-Sp1	460	74
**G0052**	β-D-GlcA-Sp	988	282
**G0053**	Gal-β-1,4-(6S)GlNAc-β-Sp	431	86
**G0054**	GlcNAc-α-1,3-(Glc-α-1,2-Glc-α-1,2)-Gal-α-1,3-Glc-α-Sp	208	43
**G0055**	Gal-β-1,3-GalNAc-β-1,4-(Neu5Gc-α-2,3)-Gal-β-1,4-Glc-β-Sp1	222	14
**G0056**	Sisomicin sulfate	1,583	178
**G0057**	GalNAc-α-1,3-(Fuc-α-1,2)-Gal-β-[blood A antigen trisaccharide]-Sp1	468	47
**G0058**	Fuc-α-1,2-Gal-β-1,4-GlcNAc-β-[blood H antigen trisaccharide]-Sp1	337	25
**G0059**	Gal-α-1,3-(Fuc-α-1,2)-Gal-β-[blood B antigen trisaccharide]-Sp1	359	23
**G0060**	Fuc-α-1,2-Gal-β-1,3-GlcNAc-β-1,3-Gal-β-1,4-Glc-β [LNFP I]-Sp1	737	83
**G0061**	Fuc-α-1,2-Gal-β-1,4-Glc-β-[blood H antigen trisaccharide]-Sp1	313	39
**G0062**	Gal-α-1,3-(Fuc-α-1,2)-Gal-β-1,4-Glc-β-[blood B antigen tetrasaccharide]-Sp1	213	15
**G0063**	(Fuc-α-1,2)-Gal-β-1,4-(Fuc-α-1,3)-GlcNAc-β-[Lewis Y]-Sp1	300	21
**G0064**	(Fuc-α-1,2)-Gal-β-1,3-(Fuc-α-1,4)-GlcNAc-β-[Lewis B]-Sp1	167	24
**G0065**	Gal-β-1,3-(Fuc-α-1,4)-GlcNAc-β-1,3-Gal-β-1,4-(Fuc-α-1,4)-Glc-β-[Lewis A]-Sp1	194	23
**G0066**	Gal-β-1,3-GalNAc-β-Sp1	148	26
**G0067**	Gal-β-1,3-(Neu5Ac-α-2,6)-GalNAc-β-Sp	355	36
**G0068**	Neu5Ac-α-2,6-Gal-β-1,3-GalNAc-β-Sp	468	25
**G0069**	Neu5Ac-α-2,6-Gal-β-1,3-(Neu5Ac-α-2,6)-GalNAc-β-Sp	423	46
**G0070**	Neu5Ac-α-2,3-Gal-β-1,3-(Neu5Ac-α-2,6)-GalNAc-β-Sp	294	29
**G0071**	Neu5Ac-α-2,6-(Neu5Ac-α-2,3)-Gal-β-1,3-GalNAc-β-Sp	422	30
**G0072**	GalNAc-β-1,4-(Neu5Ac-α-2,3)-Gal-β-1,4-Glc-β-[GM2]-Sp	421	39
**G0073**	GalNAc-β-1,4-(Neu5Ac-α-2,8-Neu5Ac-α-2,3)-Gal-β-1,4-Glc-β-[GD2]-Sp	35	6
**G0074**	Gal-α-1,4-Gal-β-1,4-GlcNAc-β-Sp1	324	22
**G0075**	β-D-Rha-Sp	617	27
**G0076**	Glc-α-1,4-Glc-β-Sp1	1,238	28
**G0077**	Glc-α-1,6-Glc-α-1,4-Glc-β-Sp1	664	69
**G0078**	Maltotriose-β-Sp1	854	10
**G0079**	Glc-α-1,6-Glc-α-1,6-Glc-β-Sp1	794	16

**G0080**	Maltotetraose-β-Sp1	2,577	352
**G0081**	GlcNAc-α-1,4-GlcA-β-1,4-GlcNAc-α1,4-GlcA-β-Sp	275	53
**G0082**	Maltohexaose-β-Sp1	7,524	1,977
**G0083**	Maltoheptaose-β-Sp1	8,132	1,811
**G0084**	Acarbose-β-Sp1	730	94
**G0085**	D-Pentamannuronic acid-β-Sp1	593	35
**G0086**	L-Pentaguluronic acid-β-Sp1	801	39
**G0087**	D-Cellose-β-Sp1	1,165	83
**G0088**	Gal-α-1,3-Gal-β-Sp1	1,048	169
**G0089**	β-1,4-Xylotetrose-Sp1	452	61
**G0090**	Chitin-trisaccharide-Sp1	368	64
**G0091**	KDN-α-2,8-Neu5Ac-α-2,3-Gal-β-1,4-Glc-β-Sp	127	25
**G0092**	Neu5Ac-α-2,8-Neu5Gc-α-2,3-Gal-β-1,4-Glc-β-Sp	242	40
**G0093**	Neu5Ac-α-2,8-Neu5Ac-α-2,8-Neu5Ac-α-2,3-Gal-β-1,4-Glc-β-Sp3	85	38
**G0094**	Neu5Ac-a-2,8-Neu5Ac-a-2,6-Gal-b-1,4-Glc-Sp5	25	10
**G0095**	Gal-β-1,3-GalNAc-β-1,4-(Neu5Ac-α-2,3)-Gal-β-1,4-Glc-β-Sp1	175	14
**G0096**	Gentamicin sulfate	349	144
**G0097**	Kanamycin sulfate	870	188
**G0098**	Geneticin disulfate salt (G418)	146	29
**G0099**	Neomycin trisulfate	907	95
**G0100**	SGP	285	20
**N010**	Man-α-1,6-(Man-α-1,3-)Man-α-1,6-(GlcNAc-β-1,2-Man-α-1,3-)Man-β-1,4-GlcNAc-β-1,4-GlcNAc-Sp5	1,142	117
**N011**	Man-α-1,6-(Man-α-1,3-)Man-α-1,6-(Gal-β-1,4-GlcNAc-β-1,2-Man-α-1,3-)Man-β-1,4-GlcNAc-β-1,4-GlcNAc-Sp5	1,148	174
**N012**	Man-α-1,6-(Man-α-1,3-)Man-α-1,6-(Neu5Ac-α-2,3-Gal-β-1,4-GlcNAc-β-1,2-Man-α-1,3-)Man-β-1,4-GlcNAc-β-1,4-GlcNAc-Sp5	944	206
**N013**	Man-α-1,6-(Man-α-1,3-)Man-α-1,6-(Neu5Ac-α-2,6-Gal-β-1,4-GlcNAc-β-1,2-Man-α-1,3-)Man-β-1,4-GlcNAc-β-1,4-GlcNAc-Sp5	989	194
**N014**	Man-α-1,6-(Man-α-1,3-)Man-α-1,6-[Gal-β-1,4-(Fuc-α-1,3-)GlcNAc-β-1,2-Man-α-1,3-]Man-β-1,4-GlcNAc-β-1,4-GlcNAc-Sp5	1,129	151
**N015**	Man-α-1,6-(Man-α-1,3-)Man-α-1,6-[Neu5Ac-α-2,3-Gal-β-1,4-(Fuc-α-1,3-)GlcNAc-β-1,2- Man-α-1,3-]Man-β-1,4-GlcNAc-β-1,4-GlcNAc-Sp5	562	61
**N020**	GlcNAc-β-1,2-Man-α-1,3-Man-β-1,4-GlcNAc-β-1,4-GlcNAc-Sp5	528	69
**N021**	Gal-β-1,4-GlcNAc-β-1,2-Man-α-1,3-Man-β-1,4-GlcNAc-β-1,4-GlcNAc-Sp5	456	79
**N022**	Neu5Ac-α-2,3-Gal-β-1,4-GlcNAc-β-1,2-Man-α-1,3-Man-β-1,4-GlcNAc-β-1,4-GlcNAc-Sp5	165	10
**N023**	Neu5Ac-α-2,6-Gal-β-1,4-GlcNAc-β-1,2-Man-α-1,3-Man-β-1,4-GlcNAc-β-1,4-GlcNAc-Sp5	201	13
**N024**	Gal-β-1,4-(Fuc-α-1,3-)GlcNAc-β-1,2-Man-α-1,3-Man-β-1,4-GlcNAc-β-1,4-GlcNAc-Sp5	259	24
**N025**	Neu5Ac-α-2,3-Gal-β-1,4-(Fuc-α-1,3-)GlcNAc-β-1,2-Man-α-1,3-Man-β-1,4-GlcNAc-β-1,4-GlcNAc-Sp5	96	8
**N026**	Gal-α-1,3-Gal-β-1,4-GlcNAc-β-1,2-Man-α-1,3-Man-β-1,4-GlcNAc-β-1,4-GlcNAc-Sp5	342	45
**N022G**	Neu5Gc-α-2,3-Gal-β-1,4-GlcNAc-β-1,2-Man-α-1,3-Man-β-1,4-GlcNAc-β-1,4-GlcNAc-Sp5	122	7
**N023G**	Neu5Gc-α-2,6-Gal-β-1,4-GlcNAc-β-1,2-Man-α-1,3-Man-β-1,4-GlcNAc-β-1,4-GlcNAc-Sp5	238	34
**N025G**	Neu5Gc-α-2,3-Gal-β-1,4-(Fuc-α-1,3-)GlcNAc-β-1,2-Man-α-1,3-Man-β-1,4-GlcNAc-β-1,4-GlcNAc-Sp5	100	8

**N030**	Man-α-1,6-(GlcNAc-β-1,2-Man-α-1,3-)Man-β-1,4-GlcNAc-β-1,4-GlcNAc-Sp5	634	89
**N210**	GlcNAc-β-1,2-Man-α-1,6-[GlcNAc(3Ac)-β-1,2-Man-α-1,3-Man-β-1,4-GlcNAc-β-1,4-GlcNAc-Sp5	425	82
**N040**	GlcNAc-β-1,2-Man-α-1,6-Man-β-1,4-GlcNAc-β-1,4-GlcNAc-Sp5	123	9
**N041**	Gal-β-1,4-GlcNAc-β-1,2-Man-α-1,3-Man-β-1,4-GlcNAc-β-1,4-GlcNAc-Sp5	217	32
**N042**	Neu5Ac-α-2,3-Gal-β-1,4-GlcNAc-β-1,2-Man-α-1,3-Man-β-1,4-GlcNAc-β-1,4-GlcNAc-Sp5	136	5
**N043**	Neu5Ac-α-2,6-Gal-β-1,4-GlcNAc-β-1,2-Man-α-1,3-Man-β-1,4-GlcNAc-β-1,4-GlcNAc-Sp5	148	10
**N044**	Gal-β-1,4-(Fuc-α-1,3-)GlcNAc-β-1,2-Man-α-1,3-Man-β-1,4-GlcNAc-β-1,4-GlcNAc-Sp5	174	18
**N045**	Neu5Ac-α-2,3-Gal-β-1,4-(Fuc-α-1,3-)GlcNAc-β-1,2-Man-α-1,3-Man-β-1,4-GlcNAc-β-1,4-GlcNAc-Sp5	164	19
**N050**	GlcNAc-β-1,2-Man-α-1,6-(Man-α-1,3-)Man-β-1,4-GlcNAc-β-1,4-GlcNAc-Sp5	286	5
**N051**	Gal-β-1,4-GlcNAc-β-1,2-Man-α-1,6-(Man-α-1,3-)Man-β-1,4-GlcNAc-β-1,4-GlcNAc-Sp5	205	17
**N052**	Neu5Ac-α-2,3-Gal-β-1,4-GlcNAc-β-1,2-Man-α-1,6-(Man-α-1,3-)Man-β-1,4-GlcNAc-β-1,4-GlcNAc-Sp5	102	5
**N053**	Neu5Ac-α-2,6-Gal-β-1,4-GlcNAc-β-1,2-Man-α-1,6-(Man-α-1,3-)Man-β-1,4-GlcNAc-β-1,4-GlcNAc-Sp	86	12
**N054**	Gal-β-1,4-(Fuc-α-1,3-)GlcNAc-β-1,2-Man-α-1,6-(Man-α-1,3-)Man-β-1,4-GlcNAc-β-1,4-GlcNAc-Sp5	128	11
**N055**	Neu5Ac-α-2,3-Gal-β-1,4-(Fuc-α-1,3-)GlcNAc-β-1,2-Man-α-1,6-(Man-α-1,3-)Man-β-1,4-GlcNAc-β-1,4-GlcNAc-Sp5	97	3
**TE001**	Neu5Ac-α-2,6-Gal-β-1,4-GlcNAc-Man-α-1,3-(Neu5Ac-α-2,6-Gal-β-1,4-GlcNAc-Man-α-1,6-)Man-β-1,4-GlcNAc-β-1,4-GlcNAc-β-Asn	84	11
**TE002**	Gal-β-1,4-GlcNAc-β-1,2-Man-α-1,3-(Gal-β-1,4-GlcNAc-β-1,2-Man-α-1,6-)Man-β-1,4-GlcNAc-β-1,4-GlcNAc-β-Asn	263	48
**TE003**	Neu5Gc-α-2,6-Gal-β-1,4-GlcNAc-β-1,2-Man-α-1,3-(Neu5Gc-α-2,6-Gal-β-1,4-GlcNAc-β-1,2-Man-α-1,6-)Man-β-1,4-GlcNAc-β-1,4-GlcNAc-β-Asn	89	25
**TE004**	Neu5Ac-α-2,3-Gal-β-1,4-GlcNAc-β-1,2-Man-α-1,3-(Neu5Ac-α-2,3-Gal-β-1,4-GlcNAc-β-1,2-Man-α-1,6-)Man-β-1,4-GlcNAc-β-1,4-GlcNAc-β-Asn	37	21
**TE005**	Neu5Gc-α-2,3-Gal-β-1,4-GlcNAc-β-1,2-Man-α-1,3-(Neu5Gc-α-2,3-Gal-β-1,4-GlcNAc-β-1,2-Man-α-1,6-)Man-β-1,4-GlcNAc-β-1,4-GlcNAc-β-Asn	31	28
**TE006**	Gal-β-1,4-(Fucα-1,3-)GlcNAc-β-1,2-Man-α-1,3-[Gal-β-1,4-(Fucα-1,3-)GlcNAc-β-1,2-Man-α-1,6-]Man-β-1,4-GlcNAc-β-1,4-GlcNAc-β-Asn	15	5
**TE007**	Gal-α-1,3-Gal-β-1,4-GlcNAc-β-1,2-Man-α-1,3-(Gal-α-1,3-Gal-β-1,4-GlcNAc-β-1,2-Man-α-1,6-)Man-β-1,4-GlcNAc-β-1,4-GlcNAc-β-Asn	101	31
**TE008**	Gal-β-1,4-(Fucα-1,3-)GlcNAc-β-1,2-Man-α-1,3-[Gal-β-1,4-(Fucα-1,3-)GlcNAc-β-1,2-Man-α-1,6-]Man-β-1,4-GlcNAc-β-1,4-GlcNAc-β-Asn	6	4
**TE009**	Neu5Ac-α-2,8-Neu5Ac-α-2,6-Gal-β-1,4-GlcNAc-β-1,2-Man-α-1,3-(Neu5Ac-α-2,8-Neu5Ac-α-2,6-Gal-β-1,4-GlcNAc-β-1,2-Man-α-1,6-)Man-β-1,4-GlcNAc-β-1,4-GlcNAc-β-Asn	7	6
**TE010**	Neu5Gc-α-2,8-Neu5Ac-α-2,6-Gal-β-1,4-GlcNAc-β-1,2-Man-α-1,3-(Neu5Gc-α-2,8-Neu5Ac-α-2,6-Gal-β-1,4-GlcNAc-β-1,2-Man-α-1,6-)Man-β-1,4-GlcNAc-β-1,4-GlcNAc-β-Asn	8	2
**TE011**	Neu5Ac-α-2,8-Neu5Gc-α-2,6-Gal-β-1,4-GlcNAc-β-1,2-Man-α-1,3-(Neu5Ac-α-2,8-Neu5Gc-α-2,6-Gal-β-1,4-GlcNAc-β-1,2-Man-α-1,6-)Man-β-1,4-GlcNAc-β-1,4-GlcNAc-β-Asn	10	5
**TE012**	Neu5Gc-α-2,8-Neu5Gc-α-2,6-Gal-β-1,4-GlcNAc-β-1,2-Man-α-1,3-(Neu5Gc-α-2,8-Neu5Gc-α-2,6-Gal-β-1,4-GlcNAc-β-1,2-Man-α-1,6-)Man-β-1,4-GlcNAc-β-1,4-GlcNAc-β-Asn	85	24
**TE013**	Neu5Ac-α-2,8-Neu5Ac-α-2,3-Gal-β-1,4-GlcNAc-β-1,2-Man-α-1,3-(Neu5Ac-α-2,8-Neu5Ac-α-2,3-Gal-β-1,4-GlcNAc-β-1,2-Man-α-1,6-)Man-β-1,4-GlcNAc-β-1,4-GlcNAc-β-Asn	12	4
**TE014**	Neu5Gc-α-2,8-Neu5Ac-α-2,3-Gal-β-1,4-GlcNAc-β-1,2-Man-α-1,3-(Neu5Gc-α-2,8-Neu5Ac-α-2,3-Gal-β-1,4-GlcNAc-β-1,2-Man-α-1,6-)Man-β-1,4-GlcNAc-β-1,4-GlcNAc-β-Asn	4	3

**TE015**	Neu5Ac-α-2,8-Neu5Gc-α-2,3-Gal-β-1,4-GlcNAc-β-1,2-Man-α-1,3-(Neu5Ac-α-2,8-Neu5Gc-α-2,3-Gal-β-1,4-GlcNAc-β-1,2-Man-α-1,6-)Man-β-1,4-GlcNAc-β-1,4-GlcNAc-β-Asn	2	2
**TE016**	Neu5Gc-α-2,8-Neu5Gc-α-2,3-Gal-β-1,4-GlcNAc-β-1,2-Man-α-1,3-(Neu5Gc-α-2,8-Neu5Gc-α-2,3-Gal-β-1,4-GlcNAc-β-1,2-Man-α-1,6-)Man-β-1,4-GlcNAc-β-1,4-GlcNAc-β-Asn	11	4
**TE017**	Neu5Ac-α-2,3-Gal-β-1,4-(Fucα-1,3-)GlcNAc-β-1,2-Man-α-1,3-[Neu5Ac-α-2,3-Gal-β-1,4-(Fucα-1,3-)GlcNAc-β-1,2-Man-α-1,6-]Man-β-1,4-GlcNAc-β-1,4-GlcNAc-β-Asn	5	2
**TE018**	Neu5Gc-α-2,3-Gal-β-1,4-(Fucα-1,3-)GlcNAc-β-1,2-Man-α-1,3-[Neu5Gc-α-2,3-Gal-β-1,4-(Fucα-1,3-)GlcNAc-β-1,2-Man-α-1,6-]Man-β-1,4-GlcNAc-β-1,4-GlcNAc-β-Asn	5	2
**TE019**	Neu5Ac-α-2,3-Gal-β-1,4-GlcNAc-β-1,3-Gal-β-1,4-GlcNAc-β-1,2-Man-α-1,3-(Neu5Ac-α-2,3-Gal-β-1,4-GlcNAc-β-1,3-Gal-β-1,4-GlcNAc-β-1,2-Man-α-1,6-)Man-β-1,4-GlcNAc-β-1,4-GlcNAc-β-Asn	6	2
**TE020**	Neu5Gc-α-2,3-Gal-β-1,4-GlcNAc-β-1,3-Gal-β-1,4-GlcNAc-β-1,2-Man-α-1,3-(Neu5Gc-α-2,3-Gal-β-1,4-GlcNAc-β-1,3-Gal-β-1,4-GlcNAc-β-1,2-Man-α-1,6-)Man-β-1,4-GlcNAc-β-1,4-GlcNAc-β-Asn	5	4
**TE021**	Neu5Ac-α-2,6-Gal-β-1,4-GlcNAc-β-1,3-(Neu5AC-α-2,6-)Gal-β-1,4-GlcNAc-β-1,2-Man-α-1,3-[Neu5Ac-α-2,6-Gal-β-1,4-GlcNAc-β-1,3-(Neu5AC-α-2,6-)Gal-β-1,4-GlcNAc-β-1,2-Man-α-1,6-]Man-β-1,4-GlcNAc-β-1,4-GlcNAc-β-Asn	4	3
**TE022**	Neu5Gc-α-2,6-Gal-β-1,4-GlcNAc-β-1,3-(Neu5GC-α-2,6-)Gal-β-1,4-GlcNAc-β-1,2-Man-α-1,3-[Neu5Gc-α-2,6-Gal-β-1,4-GlcNAc-β-1,3-(Neu5GC-α-2,6-)Gal-β-1,4-GlcNAc-β-1,2-Man-α-1,6-]Man-β-1,4-GlcNAc-β-1,4-GlcNAc-β-Asn	4	5
**TE023**	Neu5Ac-α-2,3-Gal-β-1,4-GlcNAc-β-1,3-(Neu5AC-α-2,6-)Gal-β-1,4-GlcNAc-β-1,2-Man-α-1,3-[Neu5Ac-α-2,3-Gal-β-1,4-GlcNAc-β-1,3-(Neu5AC-α-2,6-)Gal-β-1,4-GlcNAc-β-1,2-Man-α-1,6-]Man-β-1,4-GlcNAc-β-1,4-GlcNAc-β-Asn	19	7
**TE024**	GlcNAc-β-1,3-Gal-β-1,4-GlcNAc-β-1,2-Man-α-1,3-(GlcNAc-β-1,3-Gal-β-1,4-GlcNAc-β-1,2-Man-α-1,6-)Man-β-1,4-GlcNAc-β-1,4-GlcNAc-β-Asn	211	44
**TE025**	Gal-β-1,4-GlcNAc-β-1,3-Gal-β-1,4-GlcNAc-β-1,2-Man-α-1,3-(Gal-β-1,4-GlcNAc-β-1,3-Gal-β-1,4-GlcNAc-β-1,2-Man-α-1,6-)Man-β-1,4-GlcNAc-β-1,4-GlcNAc-β-Asn	56	13
**TE026**	GlcNAc-β-1,3-Gal-β-1,4-GlcNAc-β-1,3-Gal-β-1,4-GlcNAc-β-1,2-Man-α-1,3-(GlcNAc-β-1,3-Gal-β-1,4-GlcNAc-β-1,3-Gal-β-1,4-GlcNAc-β-1,2-Man-α-1,6-)Man-β-1,4-GlcNAc-β-1,4-GlcNAc-β-Asn	94	20
**TE027**	Gal-β-1,4-GlcNAc-β-1,3-Gal-β-1,4-GlcNAc-β-1,3-Gal-β-1,4-GlcNAc-β-1,2-Man-α-1,3-(Gal-β-1,4-GlcNAc-β-1,3-Gal-β-1,4-GlcNAc-β-1,3-Gal-β-1,4-GlcNAc-β-1,2-Man-α-1,6-)Man-β-1,4-GlcNAc-β-1,4-GlcNAc-β-Asn	8	6
**TE028**	GlcNAc-β-1,3-Gal-β-1,4-GlcNAc-β-1,3-Gal-β-1,4-GlcNAc-β-1,3-Gal-β-1,4-GlcNAc-β-1,2-Man-α-1,3-(GlcNAc-β-1,3-Gal-β-1,4-GlcNAc-β-1,3-Gal-β-1,4-GlcNAc-β-1,3-Gal-β-1,4-GlcNAc-β-1,2-Man-α-1,6-)Man-β-1,4-GlcNAc-β-1,4-GlcNAc-β-Asn	46	20
**TE029**	Gal-β-1,4-GlcNAc-β-1,3-Gal-β-1,4-GlcNAc-β-1,3-Gal-β-1,4-GlcNAc-β-1,3-Gal-β-1,4-GlcNAc-β-1,2-Man-α-1,3-(Gal-β-1,4-GlcNAc-β-1,3-Gal-β-1,4-GlcNAc-β-1,3-Gal-β-1,4-GlcNAc-β-1,3-Gal-β-1,4-GlcNAc-β-1,2-Man-α-1,6-)Man-β-1,4-GlcNAc-β-1,4-GlcNAc-β-Asn	7	3
**TE030**	GlcNAc-β-1,3-Gal-β-1,4-GlcNAc-β-1,3-Gal-β-1,4-GlcNAc-β-1,3-Gal-β-1,4-GlcNAc-β-1,3-Gal-β-1,4-GlcNAc-β-1,2-Man-α-1,3-(GlcNAc-β-1,3-Gal-β-1,4-GlcNAc-β-1,3-Gal-β-1,4-GlcNAc-β-1,3-Gal-β-1,4-GlcNAc-β-1,3-Gal-β-1,4-GlcNAc-β-1,2-Man-α-1,6-)Man-β-1,4-GlcNAc-β-1,4-GlcNAc-β-Asn	32	19
**TE031**	Gal-β-1,4-GlcNAc-β-1,3-Gal-β-1,4-GlcNAc-β-1,3-Gal-β-1,4-GlcNAc-β-1,3-Gal-β-1,4-GlcNAc-β-1,3-Gal-β-1,4-GlcNAc-β-1,2-Man-α-1,3-(Gal-β-1,4-GlcNAc-β-1,3-Gal-β-1,4-GlcNAc-β-1,3-Gal-β-1,4-GlcNAc-β-1,3-Gal-β-1,4-GlcNAc-β-1,3-Gal-β-1,4-GlcNAc-β-1,2-Man-α-1,6-)Man-β-1,4-GlcNAc-β-1,4-GlcNAc-β-Asn	4	2
**TE032**	GlcNAc-β-1,3-Gal-β-1,4-(Fucα-1,3-)GlcNAc-β-1,2-Man-α-1,3-[GlcNAc-β-1,3-Gal-β-1,4-(Fucα-1,3-)GlcNAc-β-1,2-Man-α-1,6-]Man-β-1,4-GlcNAc-β-1,4-GlcNAc-β-Asn	104	35

**TE033**	Gal-β-1,4-GlcNAc-β-1,3-Gal-β-1,4-(Fucα-1,3-)GlcNAc-β-1,2-Man-α-1,3-[Gal-β-1,4-GlcNAc-β-1,3-Gal-β-1,4-(Fucα-1,3-)GlcNAc-β-1,2-Man-α-1,6-]Man-β-1,4-GlcNAc-β-1,4-GlcNAc-β-Asn	11	4
**TE034**	Neu5Ac-α-2,3-Gal-β-1,4-GlcNAc-β-1,3-Gal-β-1,4-(Fucα-1,3-)GlcNAc-β-1,2-Man-α-1,3-[Neu5Ac-α-2,3-Gal-β-1,4-GlcNAc-β-1,3-Gal-β-1,4-(Fucα-1,3-)GlcNAc-β-1,2-Man-α-1,6-]Man-β-1,4-GlcNAc-β-1,4-GlcNAc-β-Asn	3	3
**TE035**	GlcNAc-β-1,3-Gal-β-1,4-GlcNAc-β-1,3-Gal-β-1,4-(Fucα-1,3-)GlcNAc-β-1,2-Man-α-1,3-[GlcNAc-β-1,3-Gal-β-1,4-GlcNAc-β-1,3-Gal-β-1,4-(Fucα-1,3-)GlcNAc-β-1,2-Man-α-1,6-]Man-β-1,4-GlcNAc-β-1,4-GlcNAc-β-Asn	15	7
**TE036**	Gal-β-1,4-GlcNAc-β-1,3-Gal-β-1,4-GlcNAc-β-1,3-Gal-β-1,4-(Fucα-1,3-)GlcNAc-β-1,2-Man-α-1,3-[Gal-β-1,4-GlcNAc-β-1,3-Gal-β-1,4-GlcNAc-β-1,3-Gal-β-1,4-(Fucα-1,3-)GlcNAc-β-1,2-Man-α-1,6-]Man-β-1,4-GlcNAc-β-1,4-GlcNAc-β-Asn	2	2
**TE037**	GlcNAc-β-1,3-Gal-β-1,4-GlcNAc-β-1,3-Gal-β-1,4-GlcNAc-β-1,3-Gal-β-1,4-(Fucα-1,3-)GlcNAc-β-1,2-Man-α-1,3-[GlcNAc-β-1,3-Gal-β-1,4-GlcNAc-β-1,3-Gal-β-1,4-GlcNAc-β-1,3-Gal-β-1,4-(Fucα-1,3-)GlcNAc-β-1,2-Man-α-1,6-]Man-β-1,4-GlcNAc-β-1,4-GlcNAc-β-Asn	7	5
**TE038**	Gal-β-1,4-GlcNAc-β-1,3-Gal-β-1,4-GlcNAc-β-1,3-Gal-β-1,4-GlcNAc-β-1,3-Gal-β-1,4-(Fucα-1,3-)GlcNAc-β-1,2-Man-α-1,3-[Gal-β-1,4-GlcNAc-β-1,3-Gal-β-1,4-GlcNAc-β-1,3-Gal-β-1,4-GlcNAc-β-1,3-Gal-β-1,4-(Fucα-1,3-)GlcNAc-β-1,2-Man-α-1,6-]Man-β-1,4-GlcNAc-β-1,4-GlcNAc-β-Asn	3	1
**TE039**	Gal-β-1,4-(Fucα-1,3-)GlcNAc-β-1,3-Gal-β-1,4-(Fucα-1,3-)GlcNAc-β-1,2-Man-α-1,3-[Gal-β-1,4-(Fucα-1,3-)GlcNAc-β-1,3-Gal-β-1,4-(Fucα-1,3-)GlcNAc-β-1,2-Man-α-1,6-]Man-β-1,4-GlcNAc-β-1,4-GlcNAc-β-Asn	4	5
**TE040**	GlcNAc-β-1,3-Gal-β-1,4-(Fucα-1,3-)GlcNAc-β-1,3-Gal-β-1,4-(Fucα-1,3-)GlcNAc-β-1,2-Man-α-1,3-[GlcNAc-β-1,3-Gal-β-1,4-(Fucα-1,3-)GlcNAc-β-1,3-Gal-β-1,4-(Fucα-1,3-)GlcNAc-β-1,2-Man-α-1,6-]Man-β-1,4-GlcNAc-β-1,4-GlcNAc-β-Asn	148	28
**TE041**	Gal-β-1,4-GlcNAc-β-1,3-Gal-β-1,4-(Fucα-1,3-)GlcNAc-β-1,3-Gal-β-1,4-(Fucα-1,3-)GlcNAc-β-1,2-Man-α-1,3-[Gal-β-1,4-GlcNAc-β-1,3-Gal-β-1,4-(Fucα-1,3-)GlcNAc-β-1,3-Gal-β-1,4-(Fucα-1,3-)GlcNAc-β-1,2-Man-α-1,6-]Man-β-1,4-GlcNAc-β-1,4-GlcNAc-β-Asn	8	3
**TE042**	Gal-β-1,4-(Fucα-1,3-)GlcNAc-β-1,3-Gal-β-1,4-(Fucα-1,3-)GlcNAc-β-1,3-Gal-β-1,4-(Fucα-1,3-)GlcNAc-β-1,2-Man-α-1,3-[Gal-β-1,4-(Fucα-1,3-)GlcNAc-β-1,3-Gal-β-1,4-(Fucα-1,3-)GlcNAc-β-1,3-Gal-β-1,4-(Fucα-1,3-)GlcNAc-β-1,2-Man-α-1,6-]Man-β-1,4-GlcNAc-β-1,4-GlcNAc-β-Asn	5	6
**TE043**	Gal-α-1,3-Gal-β-1,4-GlcNAc-β-1,3-Gal-β-1,4-GlcNAc-β-1,2-Man-α-1,3-(Gal-α-1,3-Gal-β-1,4-GlcNAc-β-1,3-Gal-β-1,4-GlcNAc-β-1,2-Man-α-1,6-)Man-β-1,4-GlcNAc-β-1,4-GlcNAc-β-Asn	18	3
**TE044**	Gal-α-1,3-Gal-β-1,4-GlcNAc-β-1,3-Gal-β-1,4-(Fucα-1,3-)GlcNAc-β-1,2-Man-α-1,3-[Gal-α-1,3-Gal-β-1,4-GlcNAc-β-1,3-Gal-β-1,4-(Fucα-1,3-)GlcNAc-β-1,2-Man-α-1,6-]Man-β-1,4-GlcNAc-β-1,4-GlcNAc-β-Asn	3	2
**TE045**	Gal-α-1,3-Gal-β-1,4-(Fucα-1,3-)GlcNAc-β-1,3-Gal-β-1,4-(Fucα-1,3-)GlcNAc-β-1,2-Man-α-1,3-[Gal-α-1,3-Gal-β-1,4-(Fucα-1,3-)GlcNAc-β-1,3-Gal-β-1,4-(Fucα-1,3-)GlcNAc-β-1,2-Man-α-1,6-]Man-β-1,4-GlcNAc-β-1,4-GlcNAc-β-Asn	4	3
**TE046**	Gal-α-1,3-Gal-β-1,4-GlcNAc-β-1,3-Gal-β-1,4-GlcNAc-β-1,3-Gal-β-1,4-GlcNAc-β-1,2-Man-α-1,3-(Gal-α-1,3-Gal-β-1,4-GlcNAc-β-1,3-Gal-β-1,4-GlcNAc-β-1,3-Gal-β-1,4-GlcNAc-β-1,2-Man-α-1,6-)Man-β-1,4-GlcNAc-β-1,4-GlcNAc-β-Asn	5	2
**TE047**	Gal-α-1,3-Gal-β-1,4-GlcNAc-β-1,3-Gal-β-1,4-GlcNAc-β-1,3-Gal-β-1,4-(Fucα-1,3-)GlcNAc-β-1,2-Man-α-1,3-[Gal-α-1,3-Gal-β-1,4-GlcNAc-β-1,3-Gal-β-1,4-GlcNAc-β-1,3-Gal-β-1,4-(Fucα-1,3-)GlcNAc-β-1,2-Man-α-1,6-]Man-β-1,4-GlcNAc-β-1,4-GlcNAc-β-Asn	6	4
**TE048**	Gal-α-1,3-Gal-β-1,4-GlcNAc-β-1,3-Gal-β-1,4-(Fucα-1,3-)GlcNAc-β-1,3-Gal-β-1,4-(Fucα-1,3-)GlcNAc-β-1,2-Man-α-1,3-[Gal-α-1,3-Gal-β-1,4-GlcNAc-β-1,3-Gal-β-1,4-(Fucα-1,3-)GlcNAc-β-1,3-Gal-β-1,4-(Fucα-1,3-)GlcNAc-β-1,2-Man-α-1,6-]Man-β-1,4-GlcNAc-β-1,4-GlcNAc-β-Asn	1	0
**TE049**	Gal-α-1,3-Gal-β-1,4-(Fucα-1,3-)GlcNAc-β-1,3-Gal-β-1,4-(Fucα-1,3-)GlcNAc-β-1,3-Gal-β-1,4-(Fucα-1,3-)GlcNAc-β-1,2-Man-α-1,3-[Gal-α-1,3-Gal-β-1,4-(Fucα-1,3-)GlcNAc-β-1,3-Gal-β-1,4-(Fucα-1,3-)GlcNAc-	9	4

	β-1,3-Gal-β-1,4-(Fucα-1,3-)GlcNAc-β-1,2-Man-α-1,6-]Man-β-1,4-GlcNAc-β-1,4-GlcNAc-β-Asn		
**TE050**	Gal-α-1,3-Gal-β-1,4-GlcNAc-β-1,3-Gal-β-1,4-GlcNAc-β-1,3-Gal-β-1,4-GlcNAc-β-1,3-Gal-β-1,4-GlcNAc-β-1,2-Man-α-1,3-(Gal-α-1,3-Gal-β-1,4-GlcNAc-β-1,3-Gal-β-1,4-GlcNAc-β-1,3-Gal-β-1,4-GlcNAc-β-1,3-Gal-β-1,4-GlcNAc-β-1,2-Man-α-1,6-)Man-β-1,4-GlcNAc-β-1,4-GlcNAc-β-Asn	2	2
**H0100**	GlcNAc-β-1,3-(GlcNAc-β-1,6-)Gal-β-1,4-Glc-Sp5	212	9
**H0101**	Gal-β-1,4-GlcNAc-β-1,3-(Gal-β-1,4-GlcNAc-β-1,6-)Gal-β-1,4-Glc-Sp5	112	14
**H0103**	Neu5Gc-α-2,6-Gal-β-1,4-GlcNAc-β-1,3-(Neu5Gc-α-2,6-Gal-β-1,4-GlcNAc-β-1,6-)Gal-β-1,4-Glc-Sp5	95	12
**H0105**	Fuc-α-1,2-Gal-β-1,4-GlcNAc-β-1,3-(Fuc-α-1,2-Gal-β-1,4-GlcNAc-β-1,6-)Gal-β-1,4-Glc-Sp5	63	8
**H0106**	Fuc-α-1,2-Gal-β-1,4-(Fuc-α-1,3-)GlcNAc-β-1,3-[Fuc-α-1,2-Gal-β-1,4-(Fuc-α-1,3-)GlcNAc-β-1,6-]Gal-β-1,4-Glc-Sp5	39	2
**H0200**	Gal-β-1,4-GlcNAc-β-1,3-(GlcNAc-β-1,6-)Gal-β-1,4-Glc-Sp5	82	9
**H0201**	Neu5Ac-α-2,6-Gal-β-1,4-GlcNAc-β-1,3-(GlcNAc-β-1,6-)Gal-β-1,4-Glc-Sp5	128	10
**H0202**	Neu5Gc-α-2,6-Gal-β-1,4-GlcNAc-β-1,3-(GlcNAc-β-1,6-)Gal-β-1,4-Glc-Sp5	129	20
**H0203**	Gal-β-1,4-(Fuc-α-1,3-)GlcNAc-β-1,3-(GlcNAc-β-1,6-)Gal-β-1,4-Glc-Sp5	93	1
**H0204**	Fuc-α-1,2-Gal-β-1,4-GlcNAc-β-1,3-(GlcNAc-β-1,6-)Gal-β-1,4-Glc-Sp5	81	15
**H0205**	Neu5Ac-α-2,6-Gal-β-1,4-GlcNAc-β-1,3-(Gal-β-1,4-GlcNAc-β-1,6-)Gal-β-1,4-Glc-Sp5	96	9
**H0207**	Gal-β-1,4-(Fuc-α-1,3-)GlcNAc-β-1,3-(Gal-β-1,4-GlcNAc-β-1,6-)Gal-β-1,4-Glc-Sp5	114	6
**H0208**	Fuc-α-1,2-Gal-β-1,4-GlcNAc-β-1,3-(Gal-β-1,4-GlcNAc-β-1,6-)Gal-β-1,4-Glc-Sp5	123	10
**H0209**	Fuc-α-1,2-Gal-β-1,4-(Fuc-α-1,3-)GlcNAc-β-1,3-(GlcNAc-β-1,6-)Gal-β-1,4-Glc-Sp5	95	13
**H0210**	Fuc-α-1,2-Gal-β-1,4-(Fuc-α-1,3-)GlcNAc-β-1,3-[Gal-β-1,4-(Fuc-α-1,3-)GlcNAc-β-1,6-]Gal-β-1,4-Glc-Sp5	150	31
**H0300**	GlcNAc-β-1,3-(Gal-β-1,4-GlcNAc-β-1,6-)Gal-β-1,4-Glc-Sp5	166	12
**H0301**	GlcNAc-β-1,3-(Neu5Ac-α-2,6-Gal-β-1,4-GlcNAc-β-1,6-)Gal-β-1,4-Glc-Sp5	132	23
**H0303**	GlcNAc-β-1,3-[Gal-β-1,4-(Fuc-α-1,3-)GlcNAc-β-1,6-]Gal-β-1,4-Glc-Sp5	85	11
**H0304**	Fuc-α-1,2-GlcNAc-β-1,3-(Gal-β-1,4-GlcNAc-β-1,6-)Gal-β-1,4-Glc-Sp5	92	9
**H0305**	Gal-β-1,4-GlcNAc-β-1,3-(Neu5Ac-α-2,6-Gal-β-1,4-GlcNAc-β-1,6-)Gal-β-1,4-Glc-Sp5	68	8
**H0306**	Gal-β-1,4-GlcNAc-β-1,3-(Neu5Gc-α-2,6-Gal-β-1,4-GlcNAc-β-1,6-)Gal-β-1,4-Glc-Sp5	94	13
**H0307**	Gal-β-1,4-GlcNAc-β-1,3-[Gal-β-1,4-(Fuc-α-1,3-)GlcNAc-β-1,6-]Gal-β-1,4-Glc-Sp5	94	11
**H0400**	Gal-β-1,4-Glc-Sp	1,044	110
**H0402**	GalNAc-β-1,3-Gal-β-1,4-Glc-Sp	270	50
**H0403**	Neu5Ac-α-2,3-Gal-β-1,4-Glc-Sp	468	37
**H0404**	Neu5Gc-α-2,3-Gal-β-1,4-Glc-Sp	396	38
**H0405**	Neu5Ac-α-2,6-Gal-β-1,4-Glc-Sp	412	29
**H0406**	Neu5Gc-α-2,6-Gal-β-1,4-Glc-Sp	426	46
**H0407**	Gal-α-1,3-Gal-β-1,4-Glc-Sp	271	26
**H0408**	Neu5Ac-α-2,8-Neu5Ac-α-2,3-Gal-β-1,4-Glc-Sp	107	42
**H0409**	Neu5Ac-α-2,8-Neu5Ac-α-2,6-Gal-β-1,4-Glc-Sp	148	35
**H0410**	Neu5Ac-α-2,3-Gal-α-1,3-Gal-β-1,4-Glc-Sp	191	32
**H0411**	Neu5Ac-α-2,6-Gal-α-1,3-Gal-β-1,4-Glc-Sp	281	50

**H0500**	Gal-α-1,4-Gal-β-1,4-Glc-Sp5	260	19
**H0503**	GalNAc-β-1,3-Gal-α-1,4-Gal-β-1,4-Glc-Sp5	164	6
**H0504**	Gal-β-1,3-GalNAc-β-1,3-Gal-α-1,4-Gal-β-1,4-Glc-Sp5	110	7
**H0505**	Fuc-α-1,2-Gal-β-1,3-GalNAc-β-1,3-Gal-α-1,4-Gal-β-1,4-Glc-Sp5	97	8
**H0600**	Gal-β-1,4-GlcNAc-β-1,3-Gal-β-1,4-Glc-Sp5	103	14
**H0601**	Gal-β-1,4-(Fuc-α-1,3-)GlcNAc-β-1,3-Gal-β-1,4-Glc-Sp5	153	9
**H0602**	Fuc-α-1,2-Gal-β-1,4-GlcNAc-β-1,3-Gal-β-1,4-Glc-Sp5	129	13
**H0603**	GlcNAc-β-1,3-Gal-β-1,4-GlcNAc-β-1,3-Gal-β-1,4-Glc-Sp5	170	14
**H0604**	Neu5Ac-α-2,3-Gal-β-1,4-GlcNAc-β-1,3-Gal-β-1,4-Glc-Sp5	116	20
**H0606**	Gal-β-1,4-GlcNAc-β-1,3-(Neu5Ac-α-2,6-)Gal-β-1,4-Glc-Sp5	160	8
**H0608**	Fuc-α-1,2-Gal-β-1,4-(Fuc-α-1,3-)GlcNAc-β-1,3-Gal-β-1,4-Glc-Sp5	144	7
**H0609**	Neu5Ac-α-2,3-Gal-β-1,4-(Fuc-α-1,3-)GlcNAc-β-1,3-Gal-β-1,4-Glc-Sp5	166	15
**H0610**	GlcNAc-β-1,3-Gal-β-1,4-(Fuc-α-1,3-)GlcNAc-β-1,3-Gal-β-1,4-Glc-Sp5	279	24
**H0700**	Gal-β-1,4-GlcNAc-β-1,3-Gal-β-1,4-GlcNAc-β-1,3-Gal-β-1,4-Glc-Sp5	103	13
**H0701**	GlcNAc-β-1,3-Gal-β-1,4-GlcNAc-β-1,3-Gal-β-1,4-GlcNAc-β-1,3-Gal-β-1,4-Glc-Sp5	174	21
**H0800**	Gal-β-1,4-GlcNAc-β-1,3-Gal-β-1,4-GlcNAc-β-1,3-Gal-β-1,4-GlcNAc-β-1,3-Gal-β-1,4-Glc-Sp5	115	13
**H0900**	Gal-β-1,3-GlcNAc-β-1,3-(GlcNAc-β-1,6-)Gal-β-1,4-Glc-Sp5	223	24
**L1001**	Neu5Ac-α-2,3-Gal-β-1,4-Glc-Sp5	78	21
**L1002**	Neu5Gc-α-2,3-Gal-β-1,4-Glc-Sp5	162	27
**L1003**	Kdn-α-2,3-Gal-β-1,4-Glc-Sp5	190	12
**L1011**	Neu5Ac-a-2,3-(GalNAc-b-1,4-)Gal-b-1,4-Glc-Sp5	60	17
**L1012**	Neu5Gc-a-2,3-(GalNAc-b-1,4-)Gal-b-1,4-Glc-Sp5	67	14
**L1013**	Kdn-a-2,3-(GalNAc-b-1,4-)Gal-b-1,4-Glc-Sp5	79	20
**L1021**	Neu5Ac-a-2,3-(Gal-b-1,3-GalNAc-b-1,4-)Gal-b-1,4-Glc-Sp5	58	14
**L1022**	Neu5Gc-a-2,3-(Gal-b-1,3-GalNAc-b-1,4-)Gal-b-1,4-Glc-Sp5	81	7
**L1023**	Kdn-a-2,3-(Gal-b-1,3-GalNAc-b-1,4-)Gal-b-1,4-Glc-Sp5	173	32
**L1201**	Neu5Ac-α-2,8-Neu5Ac-α-2,3-Gal-β-1,4-Glc-Sp5	83	24
**L1202**	Neu5Gc-α-2,8-Neu5Ac-α-2,3-Gal-β-1,4-Glc-Sp5	136	16
**L1203**	Kdn-α-2,8-Neu5Ac-α-2,3-Gal-β-1,4-Glc-Sp5	117	13
**L1204**	Neu5Ac-α-2,8-Neu5Gc-α-2,3-Gal-β-1,4-Glc-Sp5	64	29
**L1205**	Neu5Gc-α-2,8-Neu5Gc-α-2,3-Gal-β-1,4-Glc-Sp5	70	25
**L1206**	Kdn-α-2,8-Neu5Gc-α-2-,3-Gal-β-1,4-Glc-Sp5	65	27
**L1207**	Neu5Ac-α-2,8-Kdn-α-2,3-Gal-β-1,4-Glc-Sp5	58	21
**L1209**	Kdn-α-2,8-Kdn-α-2,3-Gal-β-1,4-Glc-Sp5	90	23
**L1211**	Neu5Ac-α-2,8-Neu5Ac-α-2,3-(GalNAc-b-1,4-)Gal-β-1,4-Glc-Sp5	39	18
**L1212**	Neu5Gc-α-2,8-Neu5Ac-α-2,3-(GalNAc-b-1,4-)Gal-β-1,4-Glc-Sp5	49	20
**L1213**	Kdn-α-2,8-Neu5Ac-α-2,3-(GalNAc-b-1,4-)Gal-β-1,4-Glc-Sp5	44	19
**L1214**	Neu5Ac-α-2,8-Neu5Gc-α-2,3-(GalNAc-b-1,4-)Gal-β-1,4-Glc-Sp5	35	11
**L1215**	Neu5Gc-α-2,8-Neu5Gc-α-2,3-(GalNAc-b-1,4-)Gal-β-1,4-Glc-Sp5	32	23
**L1216**	Kdn-α-2,8-Neu5Gc-α-2,3-(GalNAc-b-1,4-)Gal-β-1,4-Glc-Sp5	38	23
**L1221**	Neu5Ac-α-2,8-Neu5Ac-α-2,3-(Gal-b-1,3-GalNAc-b-1,4-)Gal-β-1,4-Glc-Sp5	24	10

**L1222**	Neu5Gc-α-2,8-Neu5Ac-α-2,3-(Gal-b-1,3-GalNAc-b-1,4-)Gal-β-1,4-Glc-Sp5	64	23
**L1225**	Neu5Gc-α-2,8-Neu5Gc-α-2,3-(Gal-b-1,3-GalNAc-b-1,4-)Gal-β-1,4-Glc-Sp5	93	22
**L1226**	Kdn-α-2,8-Neu5Gc-α-2,3-(Gal-b-1,3-GalNAc-b-1,4-)Gal-β-1,4-Glc-Sp5	103	18
**L2000**	GlcNAc-β-1,3-Gal-β-1,4-Glc-Sp5	449	33
**L2100**	Gal-β-1,4-GlcNAc-β-1,3-Gal-β-1,4-Glc-Sp5	114	15
**L2101**	Gal-a-1,3-Gal-β-1,4-GlcNAc-β-1,3-Gal-β-1,4-Glc-Sp5	107	16
**L2102**	Gal-a-1,4-Gal-β-1,4-GlcNAc-β-1,3-Gal-β-1,4-Glc-Sp5	125	20
**L2111**	Neu5Ac-a-2,3-Gal-β-1,4-GlcNAc-β-1,3-Gal-β-1,4-Glc-Sp5	97	15
**L2112**	Neu5Gc-a-2,3-Gal-β-1,4-GlcNAc-β-1,3-Gal-β-1,4-Glc-Sp5	28	8
**L2113**	Kdn-a-2,3-Gal-β-1,4-GlcNAc-β-1,3-Gal-β-1,4-Glc-Sp5	63	11
**L2121**	Neu5Ac-a-2,8-Neu5Ac-a-2,3-Gal-β-1,4-GlcNAc-β-1,3-Gal-β-1,4-Glc-Sp5	58	19
**L2122**	Neu5Gc-a-2,8-Neu5Ac-a-2,3-Gal-β-1,4-GlcNAc-β-1,3-Gal-β-1,4-Glc-Sp5	78	15
**L2103**	Gal-β-1,4-(Fuc-α-1,3-)GlcNAc-β-1,3-Gal-β-1,4-Glc-Sp5	122	15
**L2104**	Gal-a-1,3-Gal-β-1,4-(Fuc-α-1,3-)GlcNAc-β-1,3-Gal-β-1,4-Glc-Sp5	96	16
**L2131**	Neu5Ac-a-2,3-Gal-β-1,4-(Fuc-α-1,3-)GlcNAc-β-1,3-Gal-β-1,4-Glc-Sp5	112	17
**L2132**	Neu5Gc-a-2,3-Gal-β-1,4-(Fuc-α-1,3-)GlcNAc-β-1,3-Gal-β-1,4-Glc-Sp5	82	12
**L2133**	Kdn-a-2,3-Gal-β-1,4-(Fuc-α-1,3-)GlcNAc-β-1,3-Gal-β-1,4-Glc-Sp5	95	12
**L2191**		77	27
**L2192**		64	16
**L2200**	GlcNAc-β-1,3-Gal-β-1,4-GlcNAc-β-1,3-Gal-β-1,4-Glc-Sp5	103	16
**L2300**	Gal-β-1,4-GlcNAc-β-1,3-Gal-β-1,4-GlcNAc-β-1,3-Gal-β-1,4-Glc-Sp5	90	15
**L2301**	Gal-a-1,3-Gal-β-1,4-GlcNAc-β-1,3-Gal-β-1,4-GlcNAc-β-1,3-Gal-β-1,4-Glc-Sp5	78	14
**L2302**	Gal-a-1,4-Gal-β-1,4-GlcNAc-β-1,3-Gal-β-1,4-GlcNAc-β-1,3-Gal-β-1,4-Glc-Sp5	81	14
**L2311**	Neu5Ac-a-2,3-Gal-β-1,4-GlcNAc-β-1,3-Gal-β-1,4-GlcNAc-β-1,3-Gal-β-1,4-Glc-Sp5	66	9
**L2312**	Neu5Gc-a-2,3-Gal-β-1,4-GlcNAc-β-1,3-Gal-β-1,4-GlcNAc-β-1,3-Gal-β-1,4-Glc-Sp5	74	12
**L2303**	Gal-β-1,4-GlcNAc-β-1,3-Gal-β-1,4-(Fuc-α-1,3-)GlcNAc-β-1,3-Gal-β-1,4-Glc-Sp5	77	13
**L2304**	Gal-β-1,4-(Fuc-α-1,3-)GlcNAc-β-1,3-Gal-β-1,4-(Fuc-α-1,3-)GlcNAc-β-1,3-Gal-β-1,4-Glc-Sp5	80	16
**L2391**		74	21
**L2392**		90	10
**L2900**	Gal-β-1,3-GlcNAc-β-1,3-Gal-β-1,4-Glc-Sp5	340	49
**L2911**	Neu5Ac-a-2,3-Gal-β-1,3-GlcNAc-β-1,3-Gal-β-1,4-Glc-Sp5	127	26
**L2912**	Neu5Gc-a-2,3-Gal-β-1,3-GlcNAc-β-1,3-Gal-β-1,4-Glc-Sp5	149	24
**L2913**	Kdn-a-2,3-Gal-β-1,3-GlcNAc-β-1,3-Gal-β-1,4-Glc-Sp5	134	20
**L3100**	Gal-α-1,4-Gal-β-1,4-Glc-Sp5	150	25
**L3101**	GalNAc-β-1,3-Gal-α-1,4-Gal-β-1,4-Glc-Sp5	103	19
**L3102**	Gal-β-1,3-GalNAc-β-1,3-Gal-α-1,4-Gal-β-1,4-Glc-Sp5	100	17
**L3111**	Neu5Ac-a-2,3-Gal-β-1,3-GalNAc-β-1,3-Gal-α-1,4-Gal-β-1,4-Glc-Sp5	62	11
**L3112**	Neu5Gc-a-2,3-Gal-β-1,3-GalNAc-β-1,3-Gal-α-1,4-Gal-β-1,4-Glc-Sp5	73	18
**L3113**	Kdn-a-2,3-Gal-β-1,3-GalNAc-β-1,3-Gal-α-1,4-Gal-β-1,4-Glc-Sp5	89	30
**L3103**		98	20

**L3200**	Gal-α-1,3-Gal-β-1,4-Glc-Sp5	140	34
**L3201**	GalNAc-β-1,3-Gal-α-1,3-Gal-β-1,4-Glc-Sp5	117	13
**L3202**	Gal-β-1,3-GalNAc-β-1,3-Gal-α-1,3-Gal-β-1,4-Glc-Sp5	87	14
**L3211**	Neu5Ac-a-2,3-Gal-β-1,3-GalNAc-β-1,3-Gal-α-1,3-Gal-β-1,4-Glc-Sp5	77	16
**L3212**	Neu5Gc-a-2,3-Gal-β-1,3-GalNAc-β-1,3-Gal-α-1,3-Gal-β-1,4-Glc-Sp5	81	20
**L3213**	Kdn-a-2,3-Gal-β-1,3-GalNAc-β-1,3-Gal-α-1,3-Gal-β-1,4-Glc-Sp5	88	16
